# Neuronal subtype governs amyloid structure, cellular response, and cognitive outcome in genetically targeted APP mouse models

**DOI:** 10.1186/s13024-025-00919-9

**Published:** 2026-01-06

**Authors:** Gabriella A. Perez, Zoe Lai, George A. Edwards III, Jacob M. Dundee, Shannon N. Leahy, Chuangye Qi, Yanyan Qi, Ye-Jin Park, Tzu-Chiao Lu, M. Danish Uddin, Rong Zhao, Hui Zheng, Hongjie Li, Joanna L. Jankowsky

**Affiliations:** 1 Department of Neuroscience, Baylor College of Medicine, Houston, TX 77030 USA; 2 Department of Neuroscience, Washington University School of Medicine, St. Louis, MO 63110 USA; 3 Huffington Center on Aging, Baylor College of Medicine, Houston, TX 77030 USA; 4 Department of Molecular and Human Genetics, Baylor College of Medicine, Houston, TX 77030 USA; 5 Jan and Dan Duncan Neurological Research Institute, Texas Children’s Hospital, Houston, TX 77030 USA

**Keywords:** Neuronal subtype, GABAergic, APP, Amyloid plaque, Transgenic mouse, Heterogeneity

## Abstract

Pathological heterogeneity is increasingly appreciated in Alzheimer’s disease, yet we do not know how distinct aggregate conformations arise or influence cognitive outcomes. In an amyloid mouse model, we found that different brain regions formed structurally distinct Aβ deposits, prompting us to test whether neuronal subtypes shape aggregate conformation. To address this, we created transgenic mice expressing the same APP construct in either glutamatergic or GABAergic neurons. APP expression in GABAergic neurons resulted in diffuse plaques with high Aβ42/Aβ40 ratios and minimal gliosis, while glutamatergic expression produced neuritic plaques with activated glia. Despite similar Aβ levels, only mice with neuritic plaques exhibited cognitive deficits. These results show that the neuronal source of APP shapes Aβ plaque structure, influencing both cellular response and behavioral outcome. Our findings affirm that amyloid conformation and gliosis—rather than total Aβ load—drive disease progression, and suggest that regional differences in neuronal composition may govern vulnerability.

**Supplementary Information:**

The online version contains supplementary material available at 10.1186/s13024-025-00919-9.

## Introduction

The advent of cryo-electron microscopy (cryo-EM) has enabled high resolution visualization of amyloid proteins across multiple neurodegenerative diseases, in many cases uncovering an unexpected variety of aggregate structures arising from the same protein [1]. These conformational variants, or “strains”, appear in overlapping brain regions and cell types, yet can be associated with distinct clinical syndromes. The cellular and molecular processes which give rise to these differing fibrils and divergent pathological outcomes remain poorly understood. This growing body of atomic-level structural data begets two intertwined questions for the field: how does the same protein produce distinct fibril conformations, and how do these conformations beget distinct neurological trajectories?

Alzheimer’s disease (AD) exemplifies the structure-function conundrum in neurodegeneration. Though defined by amyloid plaques and neurofibrillary tangles, recent work recognizes marked heterogeneity in both pathology and clinical course [2]. This is especially evident in Aβ plaque diversity, where more than 10 distinct Aβ plaque types have been described in the literature. Divergent views on the importance of various amyloid deposits has prompted the use of two separate staging approaches [3, 4]. CERAD staging focuses on neuritic plaques, which evoke localized gliosis, neuritic dystrophy, and synapse loss [5]. In contrast, Thal staging considers only the regional distribution of Aβ deposits, irrespective of plaque morphology [6]. The plaques considered in each system differ structurally by their ability to bind intercalating dyes such as thioflavine. A more salient distinction is the cellular response to each deposit, as reactive microglia and astrocytes are suspected to promote disease progression [7]. If correct, we still do not understand how such neuroinflammation may then promote cognitive decline.

Unraveling the impact of specific Aβ aggregates in human tissue is complicated by the frequent co-occurrence of diffuse and neuritic plaques alongside other age-related lesions. Model systems are well suited to dissect cause-effect questions, however, past efforts to recreate AD pathology in mice have generally prioritized fibrillar deposits, often through overexpression of familial APP mutations that retain the wild-type Aβ domain [8]. Diffuse plaque models are less common and typically rely on species differences in Aβ sequence, or on familial mutations within the Aβ domain such as E22Q or the Uppsala variant, that differ from the wild-type peptide found in sporadic AD [9–12]; Pagnon de la Vega et al. [13], ). These species and sequence differences confound direct comparison as the models express different Aβ variants. As a result, we cannot discern how the same precursor protein generates distinct amyloid structures in AD patients, or what contribution each makes to disease progression.

While re-evaluating amyloid deposition in an existing transgenic mouse model, we observed that overexpression of APP resulted in region-specific Aβ pathology that closely paralleled human AD [3]: neuritic plaques predominated in the cortex, while diffuse plaques were more prominent in the striatum. We hypothesized that differences in the predominant neuronal subtype of each region—excitatory versus inhibitory—might underlie the formation of distinct plaque structures. To test this, we generated transgenic mice expressing an identical APP construct selectively in excitatory (Vglut1+) or inhibitory (Gad2+) neurons. This approach demonstrated that the neuronal source of APP strongly influenced both the biochemical composition and structural characteristics of Aβ deposits. These complementary models provided a testbed to investigate how distinct amyloid conformations arise, and what role each may play in disease progression. Together, our findings suggest that neuronal identity contributes to the structural heterogeneity of Aβ plaques, and that plaque structure, in turn, shapes disease-related dysfunction.

## Results

### Distinct plaque structures are regionally localized in an APP transgenic model

**Fig. 1:**
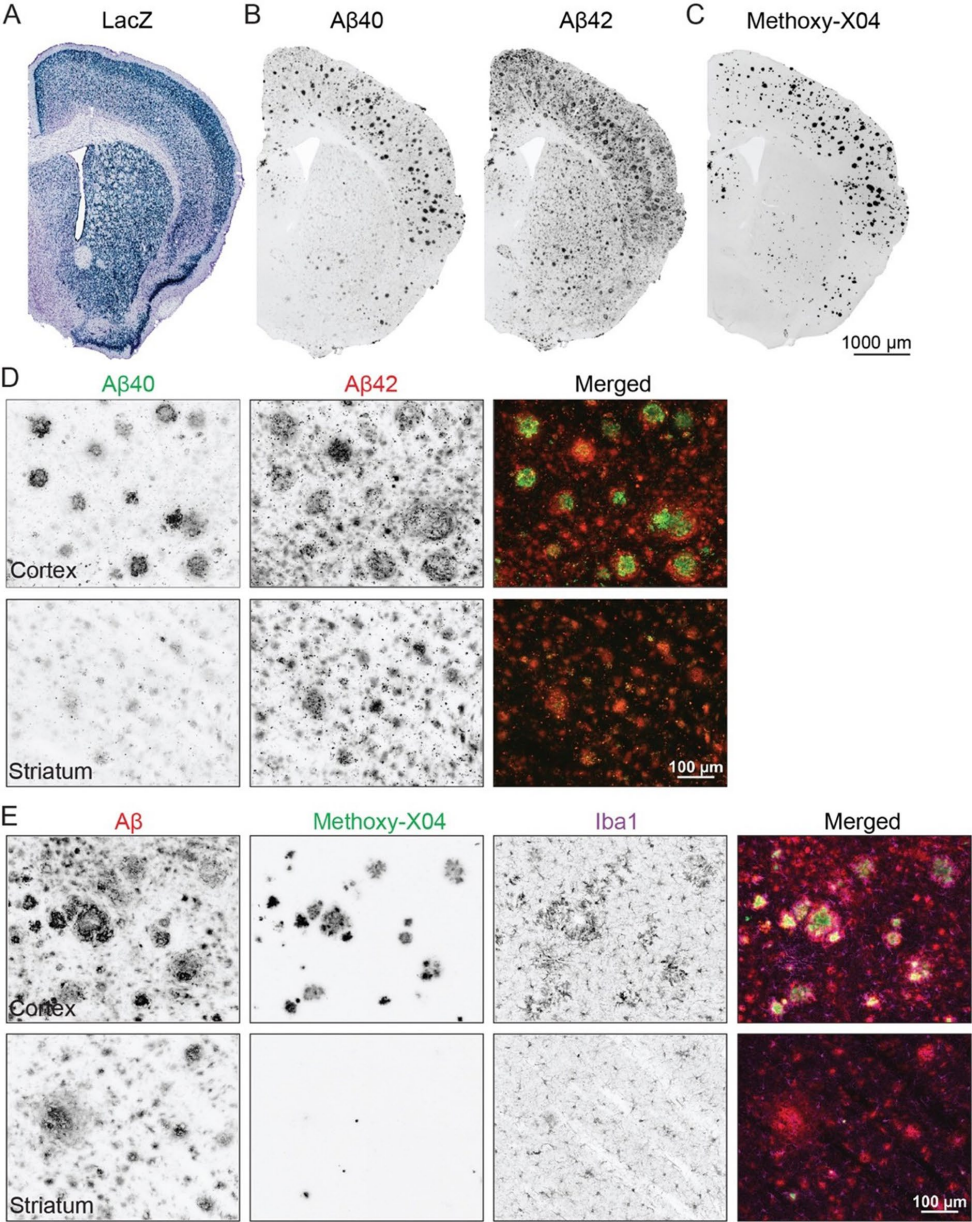
Cortex and striatum form different Aβ deposits that evoke distinct cellular reactions. (**A**) X-gal staining reveals lacZ expression controlled by the Camk2a-tTA driver when crossed to a tetO-lacZ reporter line. Strong neuronal expression appears in both cortex and striatum. Reproduced from [22]. (**B**) Aβ immunostaining reveals amyloid deposits in both cortex and striatum of Camk2a-tTA;tetO-APP mice. Immunostaining with C-terminal end-specific Aβ40 and Aβ42 antibodies reveals that Aβ42 predominates in striatum, while cortex accumulates both peptides. (**C**) Cortical deposits are strongly stained by methoxy-X04; this dye identifies only rare Aβ deposits in striatum. (**D**) High magnification images show overlap of Aβ40 (green) and Aβ42 (red) plaques in cortex, while Aβ42 signal dominates in striatal deposits. (**E**) High magnification images show reactive microglia (IBA1, magenta) surrounding X04-positive (green) plaques immunostained for Aβ (red) in cortex, with little evidence of microgliosis near X04-negative Aβ plaques in striatum. All images were taken from tissue sections of a 12 mo Camk2a-tTA;tetO-APP mouse, images in panels **B**-**C** were taken from adjacent sections. Images in scale bar = 1 mm (**A**-**C**), 100 μm (**D**-**E**)

In 2005, we introduced a transgenic APP mouse model that produced human Aβ under control of the Camk2a promoter [14]. High transgene expression in the cortex produced rapid thioflavin-positive plaque accumulation with neuritic damage and a strong microglial response [14–16]. We recently noticed that the mice also developed Aβ deposits in the striatum which failed to stain with thioflavin or the related dye methoxy-X04 [17], and showed no glial response (Fig. 1). Striatal deposits stained primarily with Aβ42 with little Aβ40 detected, while cortical plaques accumulated both peptides. The high 42:40 ratio, absence of thioflavin staining and gliosis are hallmarks of diffuse plaques found in subpial cortex, cerebellum, and subcortical regions of human Alzheimer’s brain complementary to the Camk2a-APP striatal localization [3, 18]. In contrast, the strong cortical X-04 staining, Aβ40, and reactive gliosis are characteristic of fibrillar or neuritic amyloid common in neocortical areas of human AD. These brain regions are composed of different neuronal subtypes that is partly captured by the broad activity of the Camk2a promoter in transgenic mice. In the cortex and hippocampus, the Camk2a promoter drives expression in glutamatergic neurons, while in the striatum, it expresses in GABAergic neurons [19–21]. That a single transgene produced contrasting amyloid morphologies in each region suggested to us that the same precursor can generate distinct Aβ deposits depending on its neuronal source.

### Genetic strategies to selectively overexpress APPswe/ind from excitatory or inhibitory neurons

**Fig. 2:**
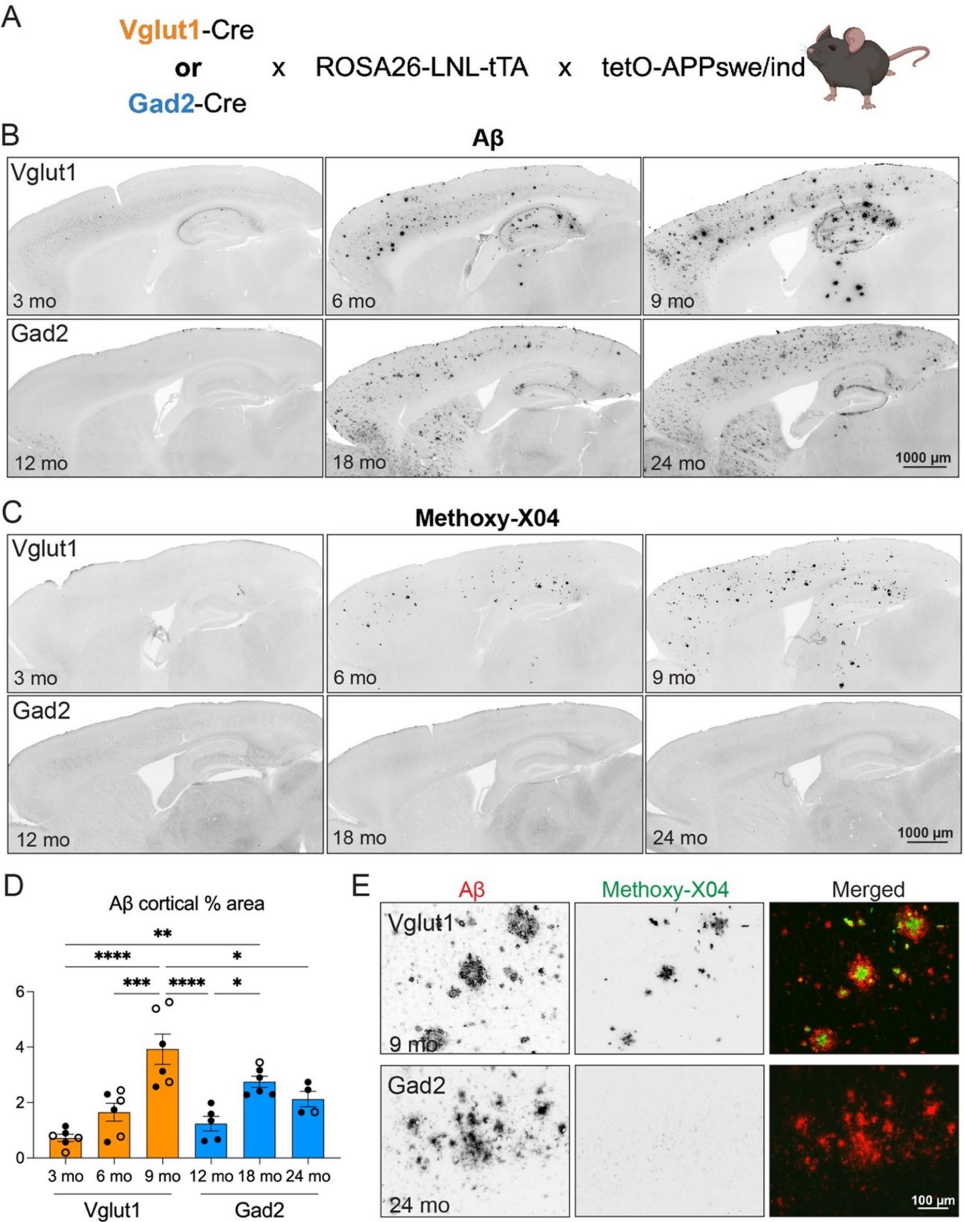
Excitatory neurons generate fibrillar amyloid plaques; inhibitory neurons produce diffuse Aβ deposits. (**A**) Breeding strategy used to express transgenic APP in excitatory (orange) or inhibitory (blue) neurons, using Vglut1-Cre or Gad2-Cre driver lines, with a Cre-dependent tTA converter line, to express tTA-responsive APPswe/ind. (**B**) Representative brain sections from Vglut1-APP mice at 3, 6, and 9 months (top row) and Gad2-APP mice at 12, 18, and 24 months (bottom row), immunostained for Aβ (human-specific clone Ab9). (**C**) Representative brain sections from Vglut1- (top row) and Gad2-APP mice (bottom row) stained for methoxy-X04 at the same ages as above. (**D**) Percent of cortical area occupied by Aβ immunostaining (mouse + human polyclonal anti-Aβ) in Vglut1- and Gad2-APP models at each age. (**E**) High magnification co-stain for Aβ (Ab9) and methoxy-X04 from cortex of Vglut1-APP at 9 months (top row) and Gad2-APP at 24 months (bottom row). *n* = 4–6 mice per group (male – closed circles, female – open circles). Data are presented as mean ± S.E.M. One-way ANOVA with Tukey’s posttest. *p < 0.05, **p < 0.01, ***p < 0.001, ****p < 0.0001. Scale bar = 1 mm (**B**, **C**), 100 μm (**e**)

We set out to test the hypothesis that neuronal source governed Aβ plaque structure by creating complementary mouse models to selectively express the same APP construct from either glutamatergic or GABAergic neurons. Our strategy used subtype-selective Cre drivers, combined with a Cre-to-tTA converter line, to express a tet-controlled APP transgene. We used Slc17a7-IRES2-Cre-D [23] for expression in glutamatergic neurons, and Gad2-IRES-Cre [24] for expression in GABAergic neurons. For each model, we crossed the Cre-to-tTA converter line ROSA26:LNL:tTA [25] with the tetracycline-responsive APP transgenic line *tetO-APP*^*swe/ind*^ line 102 [14] to generate bigenic tTA;APP mice, which were then mated with one of the two Cre driver lines. This breeding strategy separated Cre and LNL:tTA transgenes until the final intercross to avoid risk of germline LNL deletion. The resulting Cre;tTA;APP transgenic offspring overexpressed a dual mutant APP construct encoding a humanized Aβ domain (APP695swe/ind). This APProach produced two APP mouse models expressing the same form of pathogenic APP in either glutamatergic neurons (referred to as Vglut1-APP or excitatory APP mice) or GABAergic neurons (referred to as Gad2-APP or inhibitory APP mice; Fig. 2A, Supplemental Fig. 1).

### Opposing neuronal subtypes produce distinct Aβ deposits

APP overexpression produced Aβ deposits in both glutamatergic and GABAergic APP models, but at different ages corresponding to relative density of each neuronal subtype. Cortical Aβ deposits were present by 6 months (mo) in the excitatory APP mice, but did not appear until 18 mo in the inhibitory APP mice (Fig. 2B, D). Plaque load increased with time in both models to reach comparable levels at 9 and 24 mo, respectively. While immunostaining confirmed the presence of Aβ deposits in both models, only excitatory APP mice labeled with the amyloid dye methoxy-X04. Aβ plaques in Vglut1-APP mice were X04+ from the time they appeared; in contrast, cortical deposits in Gad2-APP mice did not stain for X04 at any age tested (Fig. 2C, E). This dichotomy suggests that Aβ deposits formed from inhibitory neurons were structurally distinct from those made by excitatory neurons. GABAergic neurons produced diffuse aggregates, similar to those found in the striatum of Camk2a-APP mice, while glutamatergic neurons produced fibrillar amyloid, similar to the cortical plaques of Camk2a-APP.

### Inhibitory APP mice accumulate less Aβ40 than excitatory APP mice

**Fig. 3:**
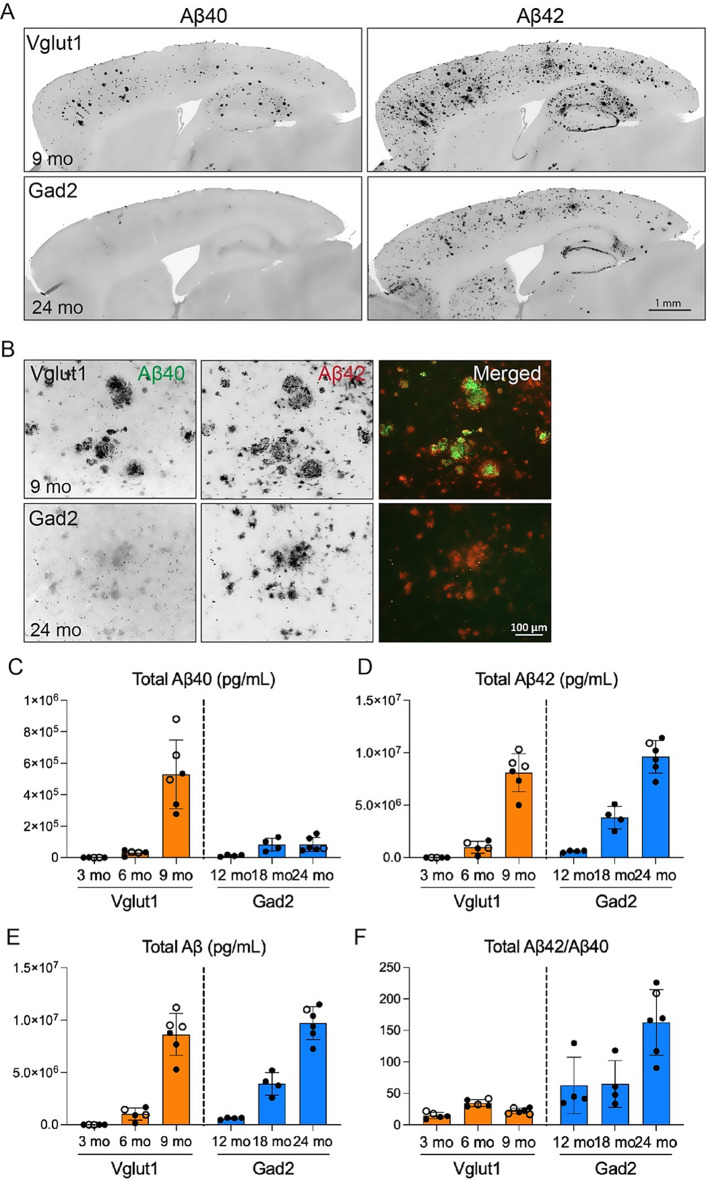
Diffuse deposits contain less Aβ40 than fibrillar plaques. (**A**) Brain sections co-immunostained for Aβ40 (left) and Aβ42 (right) in Vglut1-APP at 9 mo (top row) and Gad2-APP mice at 24 mo (bottom row). (**B**) High magnification co-immunostain for Aβ40 (green, left) and Aβ42 (red, middle), and merged channels (right) showing cortical deposits from each model. (**C**-**E**) Cortical Aβ concentration measured by immunoassay in Vglut1-APP mice at 3, 6, and 9 mo (orange) and Gad2-APP mice at 12, 18, and 24 mo (blue). Quantifications are shown for Aβ40 (**c**), Aβ42 (**D**), total Aβ (**E**), and ratio of total Aβ42:Aβ40 (**F**). *n* = 4–6 mice per group (male – closed circles, female – open circles). Data are shown as mean ± S.E.M. Scale bar = 1 mm (**A**), 100 μm (**B**)

We next examined whether the Aβ composition of plaques differed between the two models given their divergent physical structures. Cortical deposits in both models immunostained strongly for Aβ42, however, the intensity and abundance of Aβ40+ plaques was substantially greater in Vglut1-APP than in Gad2-APP mice (Fig. 3A, B). This histologic finding was confirmed by biochemical measurements of each peptide. Aβ42 was more abundant than Aβ40 by an order of magnitude in both models, and reached equivalent levels between the models at ages when plaque load was similar − 9 mo for excitatory APP mice and 24 mo for inhibitory APP (Fig. 3C–E, Supplemental Fig. 2). In contrast, Aβ40 differed markedly between excitatory and inhibitory mice at these ages, with much higher levels in the Vglut1-APP mice than in Gad2-APP. The difference in Aβ40, coupled with equivalent Aβ42, resulted in divergent 42:40 ratios for each model (Fig. 3F). That the 42:40 ratio was higher across all ages in Gad2-APP tissue than Vglut1-APP is consistent with their diffuse Aβ pathology [26, 27]. This finding suggests that the composition of Aβ aggregates contributes to their structural properties.

### APP C-terminal processing differs in excitatory and inhibitory neurons

**Fig. 4:**
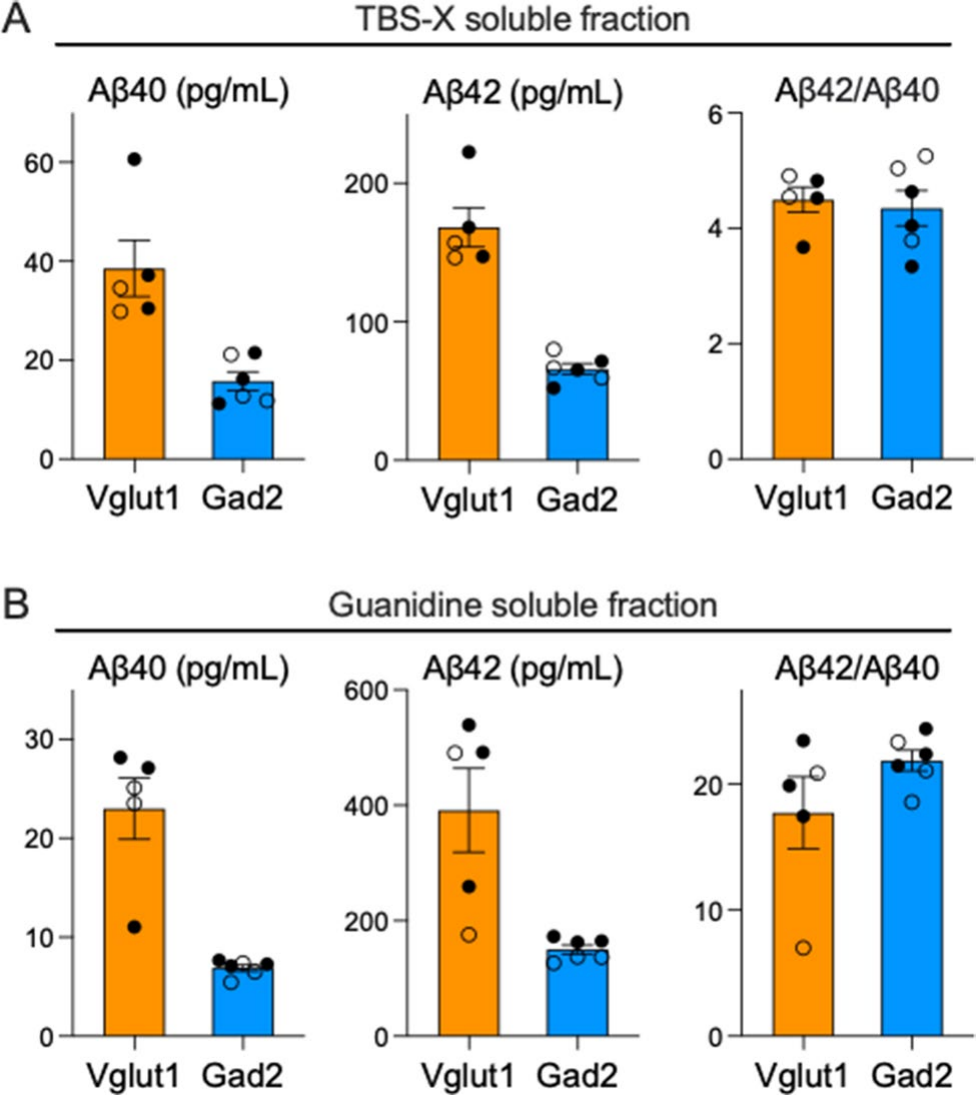
Excitatory and inhibitory neurons show comparable Aβ42:40 ratios early in life. cortical Aβ concentration measured by immunoassay in Vglut1-APP (orange) and Gad2-APP mice (blue) at 4 wk of age. Quantifications are shown for Aβ40, Aβ42, and the Aβ42:Aβ40 ratio in the TBS-X–soluble fraction (**A**) and in the guanidine-extracted pellet (**B**). *n* = 5–6 mice per group (male – closed circles, female – open circles). Data are shown as mean ± S.E.M

We asked whether subtype-specific APP processing could explain the distinct Aβ accumulation seen in excitatory versus inhibitory neurons. Although our initial analyses in aged mice hinted at differences in γ-secretase activity, these samples were dominated by insoluble Aβ even at early ages (~40:1 insoluble:soluble in Vglut1 (3 mo) and ~850:1 in Gad2-APP (12 mo)). This insoluble accumulation made it difficult to draw reliable conclusions about peptide production. To circumvent this, we examined mice at 4 weeks of age, before aggregation was limited and soluble Aβ would better reflect nascent processing. At this age, insoluble Aβ was detectable but only about twice as abundant as the soluble pool. Importantly, the Aβ42:40 ratio did not differ between Vglut1- and Gad2-APP mice (Fig. 4). These data suggest that the later divergence in Aβ accumulation arose through mechanisms other than a fixed, subtype-specific difference in γ-secretase processing.

**Fig. 5:**
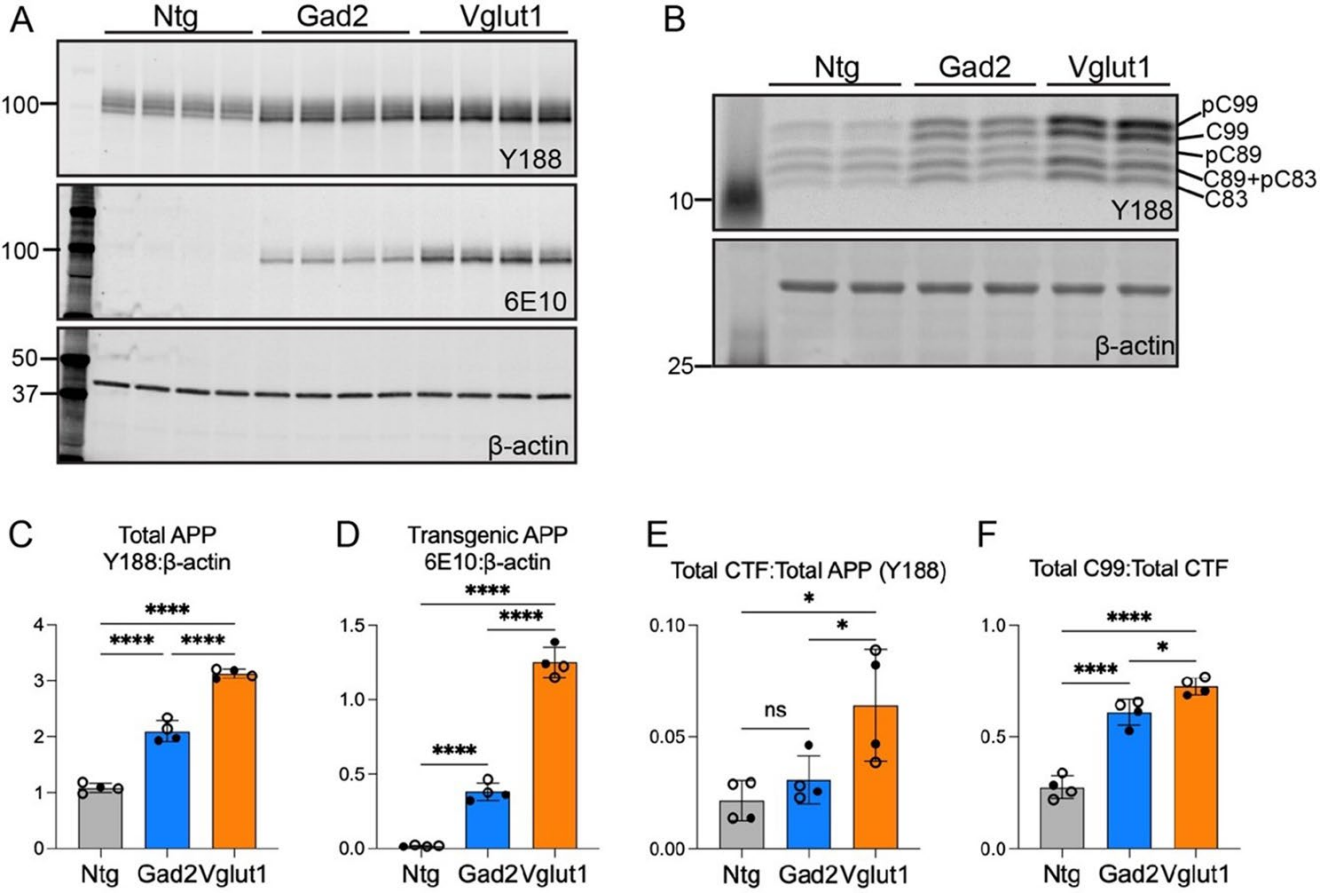
Glutamatergic neurons produce more C-terminal APP fragments than GABAergic neurons (**A**) Western blot of cortical homogenates from 3 to 4 wk old non-transgenic (Ntg), Gad2-APP, and Vglut1-APP mice probed for total full-length APP (antibody Y188; top), transgenic APP (antibody 6E10; middle), and internal control β-actin (bottom). (**B**) Western blot of cortical homogenates probed for APP C-terminal fragments (top) and internal control β-actin (bottom). (**C**-**D**) Quantification of full length APP (Y188; C) and transgenic APP (6E10; D) normalized to β-actin. (**E**) Quantification of total ctf (Y188 bands at 10–15 kD) as a fraction of total APP for each sample. (**F**) Quantification of C99 and phospho-C99 fragments as a fraction of total CTFs. *n* = 4 mice per group (male – closed circles; Female – open circles). Data are shown as mean ± S.E.M. One-way ANOVA with Tukey’s posttest. ns = non-significant, *p < 0.05, ****p < 0.0001

We next used Western blotting of cortical homogenates to test for neuronal-subtype differences in α and β-secretase cleavage than can be assessed through the pattern of APP C-terminal fragments (CTFs). We first measured full-length APP to normalize transgene expression between models. Vglut1-APP mice expressed ~1.5 fold more total APP (mouse + human) and ~3.3 fold more transgenic APP compared to Gad2-APP mice, consistent with the greater density of glutamatergic vs. GABAergic neurons in mouse cortex (Fig. 5A, C, D, Supplemental Fig. 3). We then used tricine gels to separate six APP-CTF fragments into five defined bands [28]. Relative to total full-length APP, Vglut1-APP mice produced more CTF than Gad2-APP mice, driven in part by greater β-secretase cleavage (i.e., C99, Fig. 5B, E, & F). Since most of the CTFs detected in each model originated from transgenic APP, this finding suggests that excitatory neurons process APP through α- and β secretases differently than inhibitory neurons.

We next explored whether transcription of APP processing enzymes differed between excitatory and inhibitory neurons to explain the distinct CTF and Aβ ratios. We used publicly available single-cell/single-nucleus RNA sequencing data to analyze expression of APP and its known processing enzymes (*App*, *Adam10*, *Adam17*, *Bace1*, *Bace2*, *Ncstn*, *Aph1a*, *Aph1b*, *Aph1c*, *Psen1*, *Psen2*, and *Psenen)*. We examined data for both human and mouse, including control and AD cortex and wild-type C57BL/6J isocortex [29–34] (Supplemental Figs. 4–6). We also performed snRNA-seq on cortices from Vglut1-APP mice at 9 mo, Gad2-APP mice at 24 mo, and Camk2a-APP mice at 6 mo with their respective controls (Supplemental Figs. 7–8). We measured the normalized gene expression between excitatory and inhibitory neurons in each dataset but found no significant transcriptional differences that would explain the protein-level effects we observed. This suggests one or more post-transcriptional mechanisms may be at play.

**Fig. 6:**
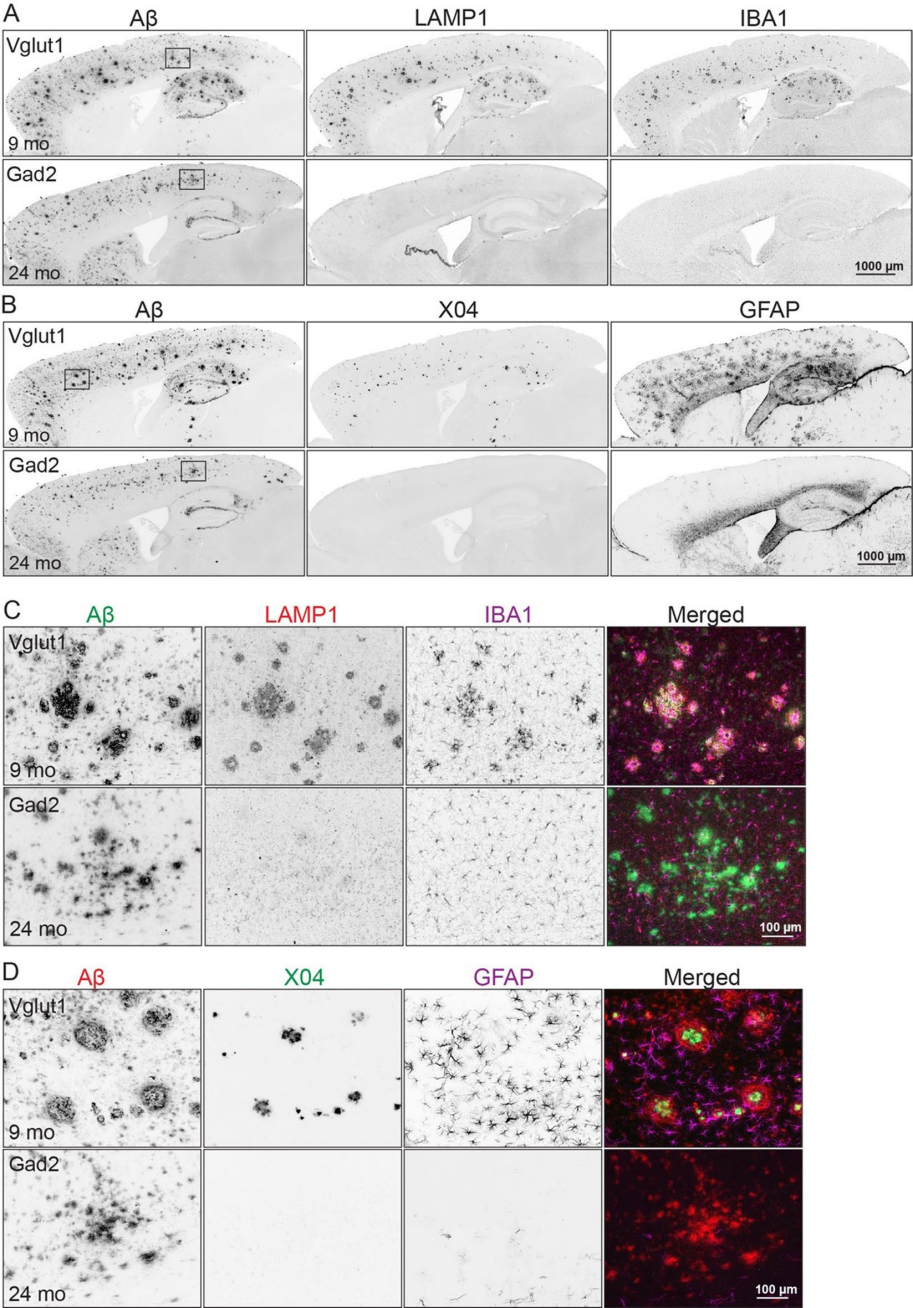
Diffuse Aβ deposits in Gad2-APP mice do not evoke neuritic damage or gliosis. (**A**) Brain sections co-immunostained for Aβ (Ab9), dystrophic neurites (LAMP1), and microglia (IBA1) in Vglut1-APP mice at 9 mo (top row) and Gad2-APP mice at 24 mo (bottom row). *n* = 3 m, 3 F Vglut1-APP; 3 m, 1 F Gad2-APP, 4–6 sections/animal, (**B**) Brain sections from Vglut1-APP (top row) and Gad2-APP (bottom row) co-stained for Aβ (Ab9), fibrillar amyloid (methoxy-X04), and reactive astrocytes (GFAP), at the same ages as above. *n* = 1 M, 1 F Vglut1-APP; 2 m, 1 F Gad2-APP, 1 section/animal. (**C**) High magnification images of cortical plaques stained for Aβ, LAMP1, and IBA1, taken from the boxed region in panel A. (**D**) High magnification images of cortical plaques stained for Aβ, methoxy-X04, and GFAP, taken from the boxed region in panel B. Scale bar = 1 mm (**a**, **B**), 100 μm (**C, D**)

**Fig. 7:**
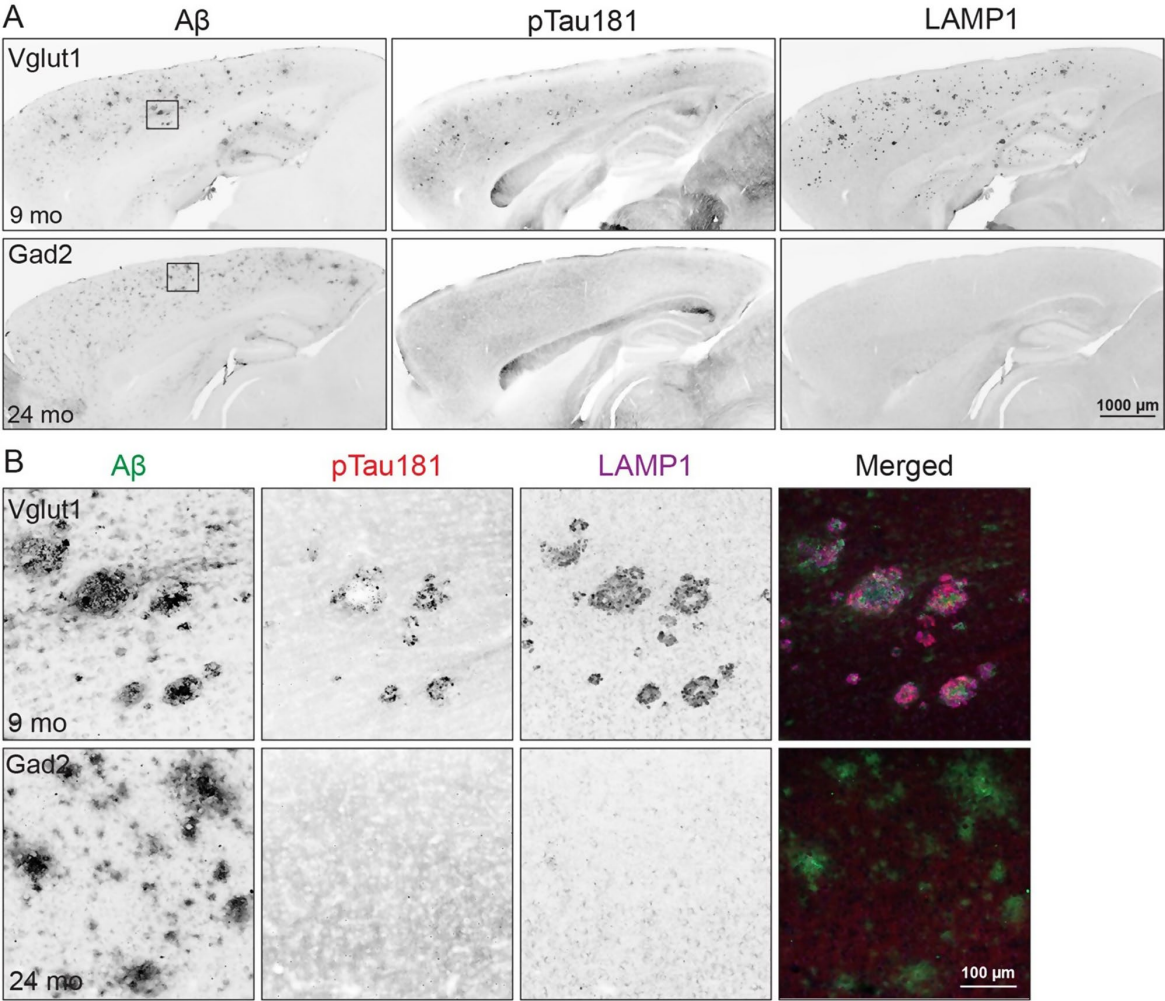
Fibrillar plaques in Vglut1-APP mice promote tau phosphorylation. (**A**) Brain sections co-immunostained for Aβ (Ab9), phosphorylated tau (pTau181), and dystrophic neurites (LAMP1) in Vglut1-APP mice at 9 mo (top row) and Gad2-APP mice at 24 mo (bottom row). *n* = 2 M, 2 F Vglut1-APP; 2 M, 2 F Gad2-APP, 2–4 sections/animal. (**B**) High magnification images of cortical plaques stained for Aβ, pTau181, and LAMP1, taken from the boxed regions in panel A. Scale bar = 1 mm (**A**), 100 μm (**B**)

**Fig. 8:**
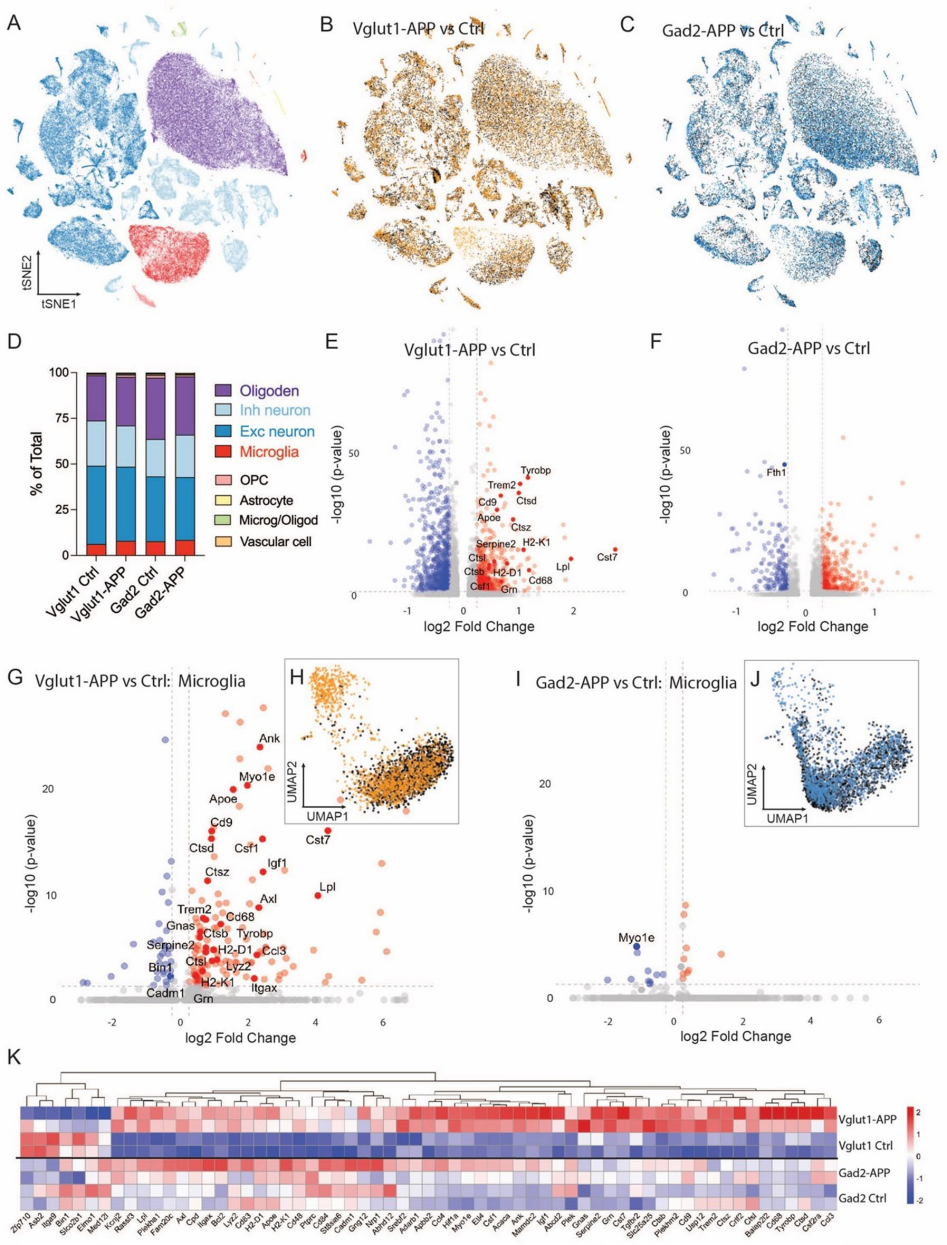
Disease-associated microglial (DAM) genes are upregulated by fibrillar plaques, but not by diffuse Aβ deposits. (**A**) t-distributed stochastic neighbor embedding (tSNE) plot of 132,154 nuclei showing 8 cell classes identified from the combined cortical snRNA sequencing data of 6 mo Camk2a-APP, 9 mo Vglut1-APP, and 24 mo Gad2-APP and age-matched controls (*n* = 4 mice/genotype). See (**D**) for color legend. (**B**) tSNE plot of 43,475 nuclei colored by genotype for 9 mo Vglut1-APP (orange) and controls (black). (**C**) tSNE plot of 42,936 nuclei colored by genotype for 24 mo Gad2-APP (blue) and controls (black). (**D**) Stacked bar graph showing total percentage of each cell type identified by genotype. (**E**) Volcano plot showing significantly up- and down-regulated genes for all cell types between Vglut1-APP and control mice. Labels identify significantly altered markers of disease-associated microglia in this and other volcano plots. Wilcoxon rank-sum test, adjusted p-value < 0.05, log_2_ FC ≥ 0.25. (**F**) Volcano plot showing significantly up- and down-regulated genes for all cell types between Gad2-APP and control mice. (**G**) Volcano plot showing significantly up- and down-regulated microglial genes between Vglut1-APP and control mice. (**H**) tSNE plot of 3076 microglial nuclei separated by genotype for Vglut1-APP (orange) and controls (black). (**I**) Volcano plot showing significantly up- and down-regulated microglial genes between Gad2-APP and control mice. (**J**) tSNE plot of 3470 microglial nuclei separated by genotype for Gad2-APP (blue) and controls (black). (**K**) Heat map visualization of all significantly altered microglial genes identified in either Vglut1- or Gad2-APP samples, relative to the average expression level in all 4 genotypes

One of the best-characterized post-translational Aβ modifications is N-terminal pyroglutamination, the target of Alzheimer’s therapeutic antibody donanemab. This modification arises through Aβ truncation by dipeptidyl peptidase 4 (DPP4), followed by cyclization of the exposed glutamate by glutaminyl cyclase (Qpct [35]; ). Pyroglutamate Aβ (Aβ-pE3) is consistently enriched in fibrillar plaques ([36–38] but see [26]), and has been proposed to occur early in plaque maturation. In the normal mouse brain, Dpp4 mRNA is highly enriched in glutamatergic neurons compared to GABAergic, whereas Qpct expression is more comparable between cell types [39]. Based on this, we asked whether Aβ-pE3 differentially marked amyloid deposits in our two models, and whether its temporal profile supported a role in shaping structural divergence. Immunostaining revealed Aβ-pE3 exclusively in fibrillar plaques of vGlut1-APP mice (Supplemental Fig. 9). Notably, however, Aβ-pE3 emerged only after plaques were detectable with methoxy-X04 and already surrounded by activated microglia, suggesting that this modification is a consequence rather than a driver of the transition to a fibrillar plaque state.

**Fig. 9:**
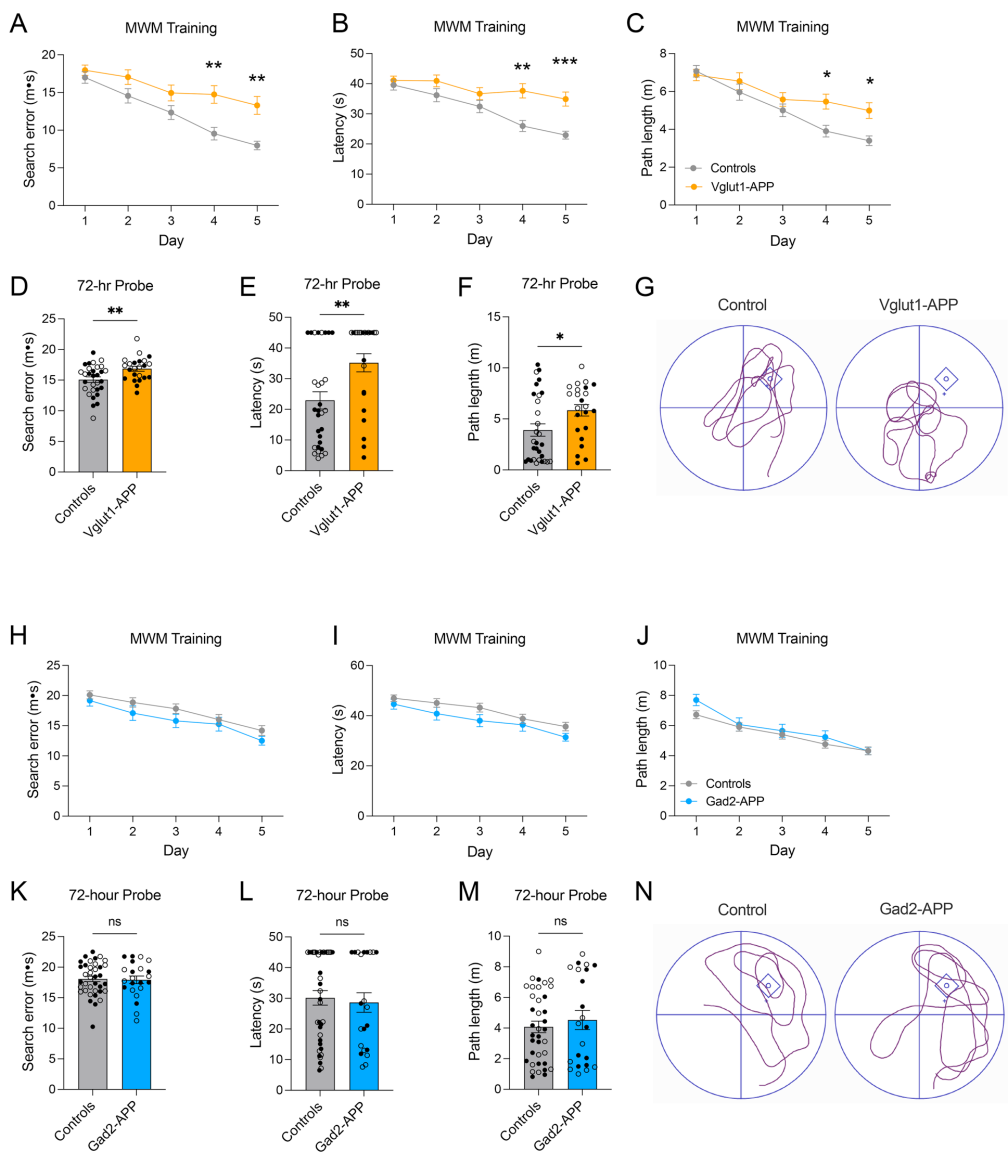
Fibrillar plaques, not diffuse deposits, are associated with spatial learning and memory deficits. (**A**-**C**) Spatial learning was assessed by water maze training at 9–12 mo in Vglut1-APP mice and age-matched sibling controls. Performance was assessed over 5 training days, averaged for 4 trials/day, as proximity to the platform (search error, a), escape latency (**B**), and path length (**C**). (**D**-**F**) Long-term memory was tested 72 hours (hr) after the final training session. Performance during probe trials was assessed as proximity (**D**), latency (**E**), and path length to the trained location (**F**). (**G**) Representative swim path traces during probe trial for control mouse (left) and Vglut1-APP mouse (right). (**H**-**J**) Water maze training for Gad2-APP mice and sibling controls was performed at 23–24 mo, and assessed by search error (**H**), escape latency (**I**), and path length (**J**). (**K**-**M**) Long-term memory was tested 72 hr later and assessed by search error (**K**), latency (**l**), and path length (**M**). (**N**) Representative swim path traces during probe trial for control mouse (left) and Gad2-APP mouse (right). *n* = 30 Vglut1 controls (wild-type or Cre+/tTA+/APP-), 23 Vglut1-APP, 37 Gad2 controls (wild-type or Cre+/tTA+/APP-), and 22 Gad2-APP. (male – closed circles; Female – open circles). Data are shown as mean ± S.E.M. Two-way repeated measure ANOVA with bonferroni posttest (**A**-**C** and **H**-**J**), unpaired two-tailed t-test (**D**, **K**), Mann-whitney two-tailed test (e, F, l, m); ns = non-significant, *p < 0.05, **p < 0.01, ***p < 0.001

### Fibrillar plaques cause gliosis and neuritic dystrophy that is absent from diffuse deposits

We next turned from what caused the plaque differences to what impact they have on the brain. Fibrillar amyloid deposits are associated with neuroinflammation and neuronal damage in human AD, whereas diffuse plaques are not [40]. Consistent with this dichotomy in human tissue, we observed that only excitatory APP mice, which developed X04+ plaques, showed microglial activation (IBA1), reactive astrogliosis (GFAP), and neuritic dystrophy (LAMP1) (Fig. 6A, B). A similar pattern of neuroinflammation had been observed around the cortical X04+ plaques in Camk2a-APP mice (Fig. 1). In contrast, Gad2-APP mice, which developed Aβ plaques that did not stain with X04, showed no evidence of neuroinflammation or neuronal damage despite equivalent Aβ load (Fig. 6A, B).

We next asked whether the neuritic damage and glial activation surrounding fibrillar plaques in Vglut1-APP mice were associated with progression from Aβ to tau pathology. We immunostained for phosphorylated tau at threonine 181 (pTau181), an early tau post-translational modification and established Alzheimer’s disease biomarker that has been reported in other mouse models with fibrillar amyloid [41–43]. By 9 months of age, pTau181 was detected around many Aβ plaques in Vglut1-APP cortex, where it colocalized with LAMP1-positive dystrophic neurites (Fig. 7). In contrast, aged Gad2-APP mice showed no evidence of pTau181 pathology, consistent with the absence of a cellular response to diffuse Aβ deposits.

We performed snRNA-seq in each model to examine these qualitative cellular responses at a quantitative transcriptional level. We included cortical samples from 9 mo Vglut1-APP, 24 mo Gad2-APP, and their respective controls, along with 6 mo Camk2a-APP and control samples (Fig. 8A–D, Supplemental Fig. 8). Across all cell types, 1392 genes were significantly altered in Vglut1-APP mice compared to age-matched controls, but far fewer genes (534) reached significance for Gad2-APP (Fig. 8E, F). Even at the pseudo-bulk level, 70 disease-associated microglia (DAM) genes were significantly altered in Vglut1-APP samples but only 3 were identified in Gad2-APP analyses. Unsupervised reclustering of microglia from the two models highlighted this difference in response, with 56 DAM genes significantly upregulated in Vglut1-APP microglia compared to a single downregulated DAM gene in Gad2-APP (Fig. 8G–J). When relative expression was visualized by heatmap for all significantly altered microglial DAM and homeostatic genes, a stark difference between Vglut1-APP and controls was apparent (Fig. 8K). This visualization also revealed an effect of age on gene expression at 24 mo that was independent of genotype, consistent with inflammaging. Viewed across the panel, the aged Gad2-APP and control samples had expression patterns that fell between the high and low extremes observed in younger Vglut1-APP mice and controls, with few genes dramatically up or downregulated between genotypes.

### Fibrillar plaques impair learning and memory; diffuse plaques do not

Finally, we investigated whether differences in plaque structure and cellular response would impact cognitive function in our transgenic models. We assessed spatial learning and memory using the Morris water maze (MWM) at ages when the two models had accumulated equal Aβ levels: 9–12 mo for Vglut1-APP and 24 mo for Gad2-APP. Each model was tested against age-matched sibling controls to account for the difference in onset. Basic locomotor and visual acuity testing did not identify any differences between genotypes or impairments with age (Supplemental Figure 10). Water maze training revealed that Vglut1-APP mice had marked deficits in spatial learning compared with controls. They swam farther and took longer to reach the target and were less accurate in their search strategy, particularly on the final days of training (Fig. 9A–C). Vglut1-APP mice also had poorer long-term memory for the trained location than controls during probe testing performed 3 d after training (Fig. 9D–F). Learning impairments were present in both sexes, however memory deficits were driven by female Vglut1-APP mice (Supplemental Figure 11). In contrast, the performance of Gad2-APP mice was indistinguishable from littermate controls, both during training and testing (Fig. 9G–L). Both sexes performed as well as controls in most measures of learning and all measures of memory (Supplemental Figure 12). Despite their advanced age, the performance of Gad2-APP and control littermates improved between days 1 and 5 of training. Nevertheless, age did have an effect on retention testing. A lower proportion of control mice reached the trained location during probe testing at 24 mo than at 9 mo, likely reflecting their slower swim speed than an impairment in memory. At 9 mo, more than twice as many Vglut1-APP mice failed to reach the trained location as controls, while at 24 mo, there was no difference between genotypes in this measure. These results show that neuritic plaques arising from glutamatergic neurons, but not diffuse deposits from GABAergic cells, contribute to impairments in spatial learning and memory.

## Discussion

Our findings reveal that the neuronal source of APP significantly influences the biochemical composition and structural properties of the resulting Aβ deposits. APP expressed from excitatory vs. inhibitory neurons gave rise to plaques with different Aβ42/Aβ40 ratios, indicating that APP processing may differ across neural subtypes. These structurally distinct aggregates elicited divergent cellular reactions from the surrounding brain tissue. Ultimately, the specific combination of Aβ composition, structure, and cellular response produced contrasting effects on cognitive function. Our findings support the concept that aggregate structure governs functional outcomes in proteinopathies. Furthermore, we propose that the neuronal source of Aβ acts as a previously unrecognized modifier of disease progression and cognitive decline.

The cognitive outcomes observed in each model support the cellular hypothesis of Alzheimer’s disease, which posits that the cellular response to protein aggregates, rather than the aggregates themselves, is central to disease pathogenesis [44–46]. While this concept may seem intuitive, our data provide functional evidence linking cellular reactions to cognitive outcomes. Fibrillar plaques derived from glutamatergic APP expression (Vglut1-APP and Camk2a-APP) provoked a pronounced local response, including neuritic dystrophy, astrocytosis, and robust microglial activation. These plaques also induced upregulation of DAM genes such as *Trem2*, *Apoe*, and *Lpl* [47, 48] and produced learning and memory deficits. In contrast, diffuse plaques in Gad2-APP mice accumulated comparable levels of Aβ over time but failed to elicit a significant cellular response or cognitive impairment. Our findings in Gad2-APP mice are consistent with extensive human clinico-pathological studies documenting diffuse Aβ accumulation in cognitive unimpaired subjects [49–52]. Although we cannot definitively conclude that the cellular response caused cognitive dysfunction, the strong correspondence between the two outcomes supports this interpretation. These findings suggest that the structural and biochemical properties of Aβ plaques—rather than total amyloid burden—critically influence the extent of glial activation and, by extension, disease progression. This connection also underlines the importance of considering aggregate–cell interactions in therapeutic strategies targeting Alzheimer’s pathology.

While our findings establish clear links between neuronal origin, plaque structure, and downstream effects, they do not fully resolve how different plaque morphologies arise from each neuronal subtype. We propose two non-exclusive mechanisms that may underlie these differences. The first involves neuronal subtype-specific processing of APP to generate distinct Aβ species. Consistent with this idea, we observed discrete patterns of APP C-terminal fragment production between the two models, suggesting differences in α- and β-secretase activity or substrate access. Yet both models produced identical proportions of Aβ42:40 early in life, suggesting that any later divergence would arise from an age-dependent shift in γ-secretase specificity. Although mRNA abundance is an imperfect proxy for protein activity [53], it remains one of the few tools available to parse expression among closely situated cell types. We acknowledge the limitation of broadly categorizing neurons into glutamatergic or GABAergic. Each term encompasses a wide range of subtypes that may contribute to and confound detection of differences in Aβ production [33, 54]. Otherwise, the glutamate-rich cerebellum would be highly susceptible instead of relatively spared.

A complementary hypothesis for plaque heterogeneity involves co-depositing proteins that influence fibril structure or cellular response. Apolipoprotein E was the first identified co-depositing modifier and remains a strong driver of fibrillar plaque formation [55–57]. More recently, mass spectrometry has identified numerous additional proteins associated with Aβ deposits [58–62], several of which alter amyloid accumulation when experimentally manipulated [63, 64]. We suspect that the microenvironment in which Aβ first aggregates—such as the local extracellular matrix or luminal conditions within endocytic compartments—may further influence its assembly pathway and contribute to structural heterogeneity. We speculate that both the neuronal source of Aβ and the molecular context into which it is released conspire to shape the resulting deposit.

We acknowledge several limitations stemming from the age difference in amyloid onset between the two models. We attribute the delayed pathology in Gad2-APP mice to the lower abundance of inhibitory neurons in cortex relative to excitatory neurons—estimated at a 4:1 glutamatergic-to-GABAergic ratio—leading to an extended lag phase before deposition in the Gad2-APP line. However, we cannot rule out the possibility that differences in plaque structure do not reflect APP processing or fibrilization, but reduced ability of aging microglia to compact amyloid. Indeed, microglia from 24 mo control mice exhibited mild inflammation associated with aging [65]. Although counterintuitive, it is possible that an attenuated microglial response in Gad2-APP animals could have been protective.

Three lines of evidence argue against immune senescence as the primary driver of structural differences. First, Tg2576 mice, which form amyloid pathology around 12–14 mo—develop fibrillar (ThioS+) plaques and a robust microglial response, indicating reactive capacity at ages only slightly younger than the Gad2-APP mice [66, 67]. Second, in the Camk2a-APP mixed model, fibrillar plaques are readily detected in the cortex, whereas diffuse plaques predominate in the striatum at the same age, suggesting that regional differences such as neuronal subtype can influence plaque structure, independent of age. Third, biochemical analysis revealed distinct patterns of APP C-terminal fragments (CTFs) between the GABAergic and glutamatergic models, indicating that these neuronal subtypes may favor different APP processing pathways. Together, these observations support a cell-type–specific basis for Aβ composition and aggregate structure.

Interpretation of our behavioral results is similarly complicated by the age disparity between models. We were initially surprised to find that older mice could successfully perform the Morris water maze task and improve with training. However, this finding is consistent with prior studies of Tg2576 mice, where aged controls (20–25 mo) demonstrated learning across trials and significant preference for the trained quadrant [68]. In our hands, 20–24-mo controls performed comparably to 9–12-mo-old animals on some metrics such as distance to the hidden platform, but poorer on others such as reduced search accuracy. This effect of age on control performance may have narrowed the dynamic range for detecting APP-related impairments. We hope to address this limitation in future studies by synchronizing amyloid onset across models using tetracycline to delay APP expression in Vglut1 mice.

Our findings suggest a novel mechanism by which neuronal composition may influence regional vulnerability. A provocative implication is that Aβ produced by GABAergic neurons may act not merely as a neutral byproduct but as a protective influence, steering aggregation toward diffuse, non-toxic deposits. Supporting this possibility, in vivo seeding studies show that amyloid strains can impose their structural properties onto the host [69, 70], and can induce diffuse pathology even in models otherwise prone to compact plaques [71, 72]. By analogy, inhibitory neurons may channel Aβ into non-reactive assemblies, diverting it from conformations that promote inflammation and neurotoxicity. Given their high APP expression and disproportionate contribution to Aβ deposition in knock-in models, GABAergic neurons may be ideally positioned to shape amyloid conformation [73–75]. In this view, the early loss of interneurons in AD could accelerate disease not only by disrupting circuits but also by eliminating this protective influence [30, 32, 76]. Exictatory:inhibitory imbalance may thus play a broader role in disease progression than previously appreciated [77]. Together, these insights provide a framework for understanding plaque heterogeneity in AD through the opposing contributions of excitatory and inhibitory neurons, and reinforce the concept that aggregate structure—not overall Aβ burden—determines cellular response and cognitive outcome.

## Methods

### Mice

*Gad2-IRES-Cre* (3’ UTR knock-in) (referred to here as inhibitory or Gad2) were purchased from The Jackson Laboratory, stock #019022 [24] and backcrossed to C57BL/6J mice at least one generation before intercrossing.

*Slc17a7-IRES2-Cre-D* (referred to here as excitatory or Vglut1) was the kind gift of Russell Ray, BCM, and are available from The Jackson Laboratory as strain #023527 [23]. Mice were backcrossed to C57BL/6J mice for > 5 generations before intercrossing.

*ROSA26:LNL:tTA* mice were purchased from The Jackson Laboratory, stock #011008 [25]. Mice were backcrossed to C57BL/6J mice for > 5 generations before intercrossing.

*tetO-APP*^swe/ind^ line 102 mice were described in [14] and are available from MMRRC as strain #34845-JAX. Mice were backcrossed to C57BL/6J mice for > 30 generations before intercrossing.

*Camk2a-tTA* mice were purchased from The Jackson Laboratory strain #007004 [20]. Mice were backcrossed to C57BL/6J for > 30 generations before intercrossing.

All lines were maintained by backcrossing on a C57BL/6J background. Both male and female animals were used for experiments except in Figs 2D (Gad2-APP 12 mo) and 3C-F (Gad2-APP 12 and 18 mo) where only male mice were available. Male mice were also used for all genotypes in snRNA-seq studies.

### Tissue harvest

Mice used for histology or in situ hybridization (ISH) were killed by pentobarbital overdose and transcardially perfused with phosphate buffered saline (PBS). For histology, the brain was removed, hemisected, and one half fixed by immersion in PBS containing 4% paraformaldehyde (PFA) at 4 °C overnight, and then cryoprotected by immersion in 30% sucrose in PBS at 4 °C until equilibrated. The remaining hemisphere was dissected to isolate the cortex and hippocampus, which were snap frozen on dry ice and stored at −80 °C until use. For ISH, one hemisphere was embedded in Tissue-Tek Cryomolds (Sakura # 4557) with Tissue-Tek O.C.T. compound (Sakura Finetek #4583) and the cryomold was quickly frozen on dry ice.

### In situ hybridization + APP immunofluorescence

A digoxigenin (DIG)-labeled mRNA antisense probes against *Gad2* and *Vglut1* was generated by reverse-transcribing mouse cDNA using a RNA DIG-labeling kit. Primer and probe sequences for *Gad2* and *Vglut1* were based on those provided by the Allen Brain Atlas (http://www.brain-map.org). Fresh-frozen 25 μm sections from O.C.T. embedded brains were mounted onto Superfrost Plus slides (#12–550-15, Fisher Scientific) and dried before use. Fluorescent ISH was performed by the BCM RNA In Situ Hybridization Core using an automated robotic platform as previously described [78]. The protocol was modified for fluorescence detection using a tyramide-FITC Plus kit to detect the DIG-labeled probe (1/50 dilution, 15 minute (min) incubation, Perkin Elmer). Following ISH detection, slides were washed in PBS and prepared for immunostaining. Sections were blocked in PBS containing 0.3% Triton X-100 (PBST) plus 10% normal goat serum for 1 hr at room temperature (RT) before overnight incubation at 4 °C with primary antibody diluted in blocking solution (1:500 mouse anti-human β-amyloid clone 6E10, BioLegend, #803002). The following day, sections were washed in PBS and incubated in secondary antibody for 2 hr at RT diluted in blocking solution (1:500 goat-anti mouse IgG1 Alexa Fluor 568, Invitrogen, Molecular Probes #A21124). Slides were washed several times with PBS and then coverslipped with ProLong Diamond Antifade mounting medium (ThermoFisher #P36970).

### Aβ immunofluorescence, imaging, and % area quantitation

Cryoprotected tissue was frozen on dry ice and sectioned sagittally at 35 µm using a freezing-sliding microtome. Sections were stored at −20 °C in cryoprotectant until use. Sections were washed several times in Tris-buffered saline (TBS) to remove cryoprotectant, incubated with 88% formic acid for 1 min at RT and then rinsed with TBS before continuing with the stain. Sections were blocked with TBS containing 0.3% Triton X-100 plus 5% normal goat serum for 1 hr at RT before overnight incubation at 4 °C in primary antibody diluted in blocking solution (1:500, rabbit anti-Aβ, ThermoFisher/Zymed, #71–5800 or 1:500 Ab9 mouse anti-human Aβ [79]) The following day, sections were washed in TBS and incubated with secondary antibody diluted in blocking solution for 2 hr at RT (1:500 goat anti-rabbit IgG Alexa Fluor 594, Invitrogen #A11037 or 1:500 goat anti-mouse IgG2a Alexa Fluor 488, Invitrogen, Molecular Probes #A21131). Sections were washed in TBS and coverslipped with ProLong Diamond antifade mounting medium (ThermoFisher #P36970).

Tiled images were acquired using a Zeiss AxioScan.Z2 at 20x magnification (Carl Zeiss AG). Exposure time and lamp intensity were consistent across all Gad2 and Camk2a sections. Vglut1 sections used for Aβ quantification were stained and imaged at a later time; however, all analysis was performed together and in the same manner. Sections used for analysis were plane-matched across animals. Aβ cortical percent area from Zymed rabbit anti-Aβ immunostaining was measured using QuPath (v0.0.5.1) open source software [80]. An ROI was drawn around the cortex and the pixel classifier threshold was used to measure Aβ total percent area. Percent area of plaque deposition was measured and averaged across 3–5 plane matched sections spanning/ranging between 0.96 mm − 1.56 mm from bregma [81].

### Aβ, LAMP1, IBA1, GFAP, pTau181, and pGlu-3 Aβ immunofluorescence ± methoxy-X04 histological staining

Free-floating sections were washed several times in TBS to remove cryoprotectant and then blocked with TBS containing 0.3% Triton X-100 plus 5–10% normal serum (goat or donkey) for 1 hr at RT before overnight incubation at 4 °C in primary antibodies diluted 1:500 in blocking solution (mouse IgG2a anti-human Aβ clone Ab9 [79]; rat anti-LAMP1, DSHB #1D4B; rabbit anti-IBA1, Wako #019–19741; mouse IgG1 anti-GFAP, Sigma #G3893; chicken anti-IBA1, Synaptic Systems #234009; and/or rabbit anti-pyroglutamate-3 Aβ, Synaptic Systems #218003). The following day, sections were washed in TBS and incubated with secondary antibody diluted 1:500 in blocking solution for 2 hr at RT (goat anti-mouse IgG2a Alexa Fluor 488 #A21131, goat anti-rat IgG Alexa Fluor 568 #A11077, goat anti-rabbit IgG Alexa Fluor 647 #A21245, goat anti-chicken IgY Alexa Fluor 647 #A21449; goat anti-mouse IgG2a Alexa Fluor 647 #A21241; and/or goat anti-rabbit IgG Alexa Fluor 568 #A11036, donkey anti-mouse IgG Alexa Fluor 488 #A21202, and/or donkey anti-rat IgG Alexa Fluor 647 #A48272 - all from Invitrogen, Molecular Probes). Sections were washed in TBS, and either mounted onto SuperFrost Plus slides and coverslipped with ProLong Diamond, or stained for methoxy-X04 before mounting by incubating for 30 second (sec) in PBS containing 40% ethanol, followed by 30 sec incubation in 100 uM methoxy-X04 (Bio-Techne, #4920) diluted in PBS/ethanol, followed by another 30 sec wash in PBS/ethanol.

Staining for phosphorylated tau 181 was performed as described above using 5% donkey serum before overnight incubation in primary antibody (1:500 rabbit anti-pTau181, Cell Signaling Technology #12885), 2 hr secondary incubation (goat anti-rabbit IgG Alexa Fluor 568), and 2 hr tertiary incubation to enhance signal (donkey anti-goat Alexa Fluor 568, Invitrogen #A11057).

### Aβ42 and Aβ40 immunofluorescence

Free-floating sections were washed several times in TBS to remove cryoprotectant, and either fixed in 4% PFA for 30 min at RT, rinsed in TBS, and incubated in 88% formic acid (SigmaAldrich #399388) for 15 min at RT, or put directly into 88% formic acid for 2.5 min at RT without post-fixation. After acid denaturation, sections were rinsed several times in TBS, and then blocked with TBS containing 0.3% Triton X-100 plus 5% normal goat serum for 1 hr at RT before overnight incubation at 4 °C in primary antibodies diluted in blocking solution (1:400, mouse IgG1 anti-Aβ42, BioLegend #805509, 1:400 rabbit anti-Aβ40, Invitrogen #44–136. The following day, sections were washed in TBS and incubated with secondary antibody diluted in blocking solution for 2 hr at RT (1:500, goat anti-mouse IgG1 Alexa Fluor 568, Invitrogen, Molecular Probes #A21124, 1:500 goat anti-rabbit IgG Alexa Fluor 488, Invitrogen, Molecular Probes #A11034). Sections were washed in TBS and coverslipped with ProLong Glass (ThermoFisher #P36982) or Prolong Diamond antifade medium.

### Soluble and insoluble Aβ extractions; Luminex Aβ40, Aβ42 measurements

Frozen tissue was sonicated using an Elite bead mill homogenizer with 1.4 mm ceramic pre-filled microtubes (Omni International, #19–617) in cold TBS containing 5 mM EDTA, 1x phosphatase inhibitor (Roche #05892970001), and 1x protease inhibitor (Roche #4906837001) at a ratio of 1 ml per 150 mg tissue weight. The homogenate was centrifuged at 100,000 g for 1 hr at 4 °C, and the supernatant collected as TBS-soluble extract. The pellet was resuspended to its original volume by pipetting in 5 M guanidine hydrochloride (GuHCl) made in 50 mM Tris-HCl, pH 6.8, and mixed by gentle rotation at RT overnight. The next day, samples were centrifuged at 16,000 g for 30 min at RT, and the supernatant collected as the insoluble (or GuHCl) fraction. All samples were stored at −80 °C until use. Aβ40 and Aβ42 were measured from TBS-soluble and insoluble extracts using the Luminex-based Milliplex MAP Human Neurodegenerative Disease Magnetic Bead Panel 4 (MilliporeSigma, #HNDG4MAG-36K), according to manufacturer’s instructions. Briefly, samples from each extraction were diluted in assay buffer (Camk2a TBS 1 mo = no dilution, 3 mo = 1:3, 6 mo = 1:5, Camk2a GuHCl 1 mo = 1:100, 3 mo = 1:1,000, 6 mo = 1: 10,000; Vglut1 TBS 3 mo = no dilution, 6 mo = 1:1, 9 mo = 1:3, 12 mo = 1:5, Vglut1 GuHCl 3 mo = 1:100, 6 mo = 1:1,000, 9 mo = 1:5,000, 12 mo = 1:10,000; Gad2 TBS 12 mo = no dilution, 18 mo = 1:3, 24 mo = 1:5, Gad2 GuHCl 12 mo = 1:1,000, 18 mo = 1: 5000, 24 mo = 1:10,000) to bring Aβ40 and Aβ42 levels within an optimized range of known standards (Aβ40 = 3.4–2500 pg/ml, Aβ42 = 27.4– 20,000 pg/ml). Plates were washed before adding 25 ul of diluted standards, quality controls, samples, Aβ40 and Aβ42 detection antibodies, and magnetic beads, and incubated overnight at 4 °C on a plate shaker at 500 rpm. The next day, plates were washed and beads incubated with 25 ul of biotinylated detection secondary antibody and streptavidin-phycoerythrin at 500 rpm for 30 min at RT. After a final wash, 100 ul of sheath fluid was added to each well and beads were resuspended on a plate shaker at 500 rpm for 5 min at RT before reading on Luminex 200 (ThermoFisher). Sample concentrations and median fluorescent intensities were calculated and analyzed using a 5-parameter logistic or spline curve-fitting method.

### Western blotting

Frozen hemicortices were homogenized by sonication in 10 volumes per weight of RIPA buffer (PBS containing 1% SDS, 5 mM EDTA, 0.5% deoxycholate, and 0.5% IGEPAL) plus phosphatase and protease inhibitors. Homogenates were centrifuged for 10 min at 16,000 g and 4 °C. The supernatant was collected and stored at −80 °C until use. Protein concentration was measured by BCA (Fisher, #23227). To detect full-length and transgenic APP, 30 ug of protein was diluted with 4x Laemmli buffer (Bio-Rad, #1610747), denatured at 95 °C for 5 min and electrophoresed on 4–15% Criterion TGX gels (Bio-Rad, #5671085). To detect APP-CTFs, 20 ug of protein was diluted with 2x Tricine SDS sample buffer (Invitrogen, #LC1676) and 10x NuPAGE reducing agent (Invitrogen, #NP0009), denatured at 85 °C for 2 min and electrophoresed on 16% Novex tris tricine gels (Invitrogen, #EC66952BOX). Proteins were transferred to nitrocellulose for 7 min at 25 V using the Trans-Blot Turbo Transfer System (Bio-Rad, #1704271). Membranes were blocked in PBS containing 5% non-fat dry milk for 1 hr at RT and probed overnight at 4 °C in blocking solution containing mouse anti-human β-amyloid clone 6E10 (1:1000), rabbit anti-APP clone Y188 (1:5000, Abcam, #ab32136), and mouse anti-β-actin clone 8H10D10 (1:5000, Cell Signaling Technology, #3700). To detect APP-CTFs, the membrane was boiled in distilled H_2_O for 15 min before blocking and probing for APP (Y188) and β-actin. Membranes were washed with PBS containing 0.1% Tween-20 and incubated for 1 hr at RT in blocking solution containing secondary antibodies 680RD goat anti-mouse IgG (Li-Cor #926–68070) and 800CW donkey anti-rabbit IgG (Li-Cor #926–32213) each diluted 1:5000. Membranes were washed again in PBS containing 0.1% Tween-20 and then imaged on a ChemiDoc MP Imaging System (Bio-Rad). Proteins were quantified using Image Lab (Bio-Rad) and normalized to β-actin.

### Human and mouse sc/snRNA sequencing data analysis

#### Preprocessing of single-cell RNA sequencing data

Raw sequencing files were demultiplexed for gene expression libraries were processed using bcl2fastq tools. After mapping sequencing reads to mm10 or hg38 reference genome by cellranger-7.1.0, the ambient RNA for each single cell was estimated and removed by Cellbender [82]. Next, doublet cells were identified using Scrublet [83] from the filtered feature barcode matrices produced by Cellbender. Cells were scored as candidate doublets by Scrublet and removed if their doublet score exceeded 0.20. Finally, remaining cells were filtered to have less than 5% of their UMIs mapped to mitochondrial genes and to express greater than 200 genes by Scanpy [84].

#### Single-cell dimensionality reduction and clustering

Single-cell analysis was performed by following the Scanpy pipeline (v1.9.8). Raw counts were log-normalized via scanpy.pp.log1p()) and subsequently used to select 3000 highly variable genes (scanpy.pp.highly_variable_genes()). Single cells were batch corrected and projected into a low-dimensional representation by single cell Variational Inference (scvi; scvitools v1.1.2) [85]. The raw counts of the highly variable genes were used to train the scVI VAE (parameters: batch_key = ‘sex’ and ‘genotype’ and ‘batch’, n_latent = 25). The 25 latent dimensions were used to determine a KNN graph with running scanpy.pp.neighbors(n_neighbors = 15) for UMAP generation (scanpy.tl.umap()) and leiden clustering (scanpy.tl.leiden (resolution = 2, 1.5 or 1)). Major cell types and Subclusters were marked and identified using well-known marker genes, quality control metrics and gene lists provided by ‘scanpy.tl.rank_genes_groups’.

### Mouse brain nuclei extraction, nuclei sorting, library preparation, and single-nucleus RNA sequencing

Frozen, frontal cortices from male 6 mo Camk2a-APP, 9 mo Vglut1-APP, and 24 mo Gad2-APP mice and controls were transcardially perfused with ice-cold PBS. After the brain was removed, one hemisphere was dissected to isolate the cortex. The hemicortex was cut in half and the rostral cortical tissue samples were snap-frozen on dry ice and stored at −80 °C until use. For each sample, 2 mouse caudal brain hemicortex tissues were pooled into one tube for single-nucleus suspensions. Single-nucleus suspensions were prepared following the protocol described previously (McLaughlin et al., 2022). Nuclei were stained by Hoechst-33342 (1:1000; > 5 min). Next, nuclei were collected using fluorescence activated cell sorting (FACS) to remove debris. The BD Aria III sorter was used for collecting nuclei. Hoechst 33342+ nuclei were collected during sorting into a 1.5 ml tube with 200 ul PBS containing 0.5% BSA as the receiving buffer (RNase inhibitor added). For each 10X Genomics run, 80k–150k nuclei were collected. Nuclei were spun down for 10 min at 950 g at 4 C, and then resuspended using 40 ul of PBS with 0.5% BSA (RNase inhibitor added). 2 ul nucleus suspension was used for counting the nuclei with hemocytometers to calculate the concentration. 20K nuclei were loaded into the 10X chip for each capture to target 10k nuclei for each run. Next, 10X Genomics sequencing libraries were prepared following the standard protocol from Chromium Next GEM Single Cell 3’ Reagent Kits v3.1 (Dual Index) (10X Genomics, #1000269) with following settings. All PCR reactions were performed using the Bio-Rad C1000 thermal cycler with 96-deep well reaction module (Bio-Rad, #1851197EDU). Cycle numbers were used as the 10X protocol recommended for cDNA amplification and sample index PCR. As per 10X protocol, 1:10 dilutions of amplified cDNA and final libraries were evaluated on Agilent TapeStation. The final library was sent to Novogene Corporation Inc. for Illumina NovaSeq PE150 S4 lane sequencing with the dual index configuration Read 1 28 cycles, Index 1 (i7) 10 cycles, Index 2 (i5) 10 cycles, and Read 2 90 cycles. A PhiX control library was spiked in at 1% concentration. The sequencing depth was about 43-60k reads per nucleus.

### Vglut1-, Gad2-, and Camk2a-APP snRNA sequencing: data processing and analysis

Raw reads demultiplexed by bcl2fastq were mapped to the modified mus musculus reference genome (mm10) with the addition of transgene sequences for Camk2a-tTA (CaMK2TTA), Gad2-Cre, Vglut1-Cre, ROSA26-LNL-tTA (R26LNL), and tetO-APP_102_ (APP695S) using CellRanger v0.8.0.1 with ‘include-introns true’ and ‘expect-cells 10,000’ parameters. Quality control filtering, variable gene selection, dimensionality reduction, and clustering for cells were conducted using the Seurat v0.5.1.0 package [86]. To filter low-quality cells, we removed cells for which less than 600 or greater than 8000 genes were detected, or cells that contained greater than 1% genes from the mitochondrial genome. Genes expressed in fewer than three cells were filtered out. The decontX v0.1.4.1 package was used to remove ambient RNAs [87]. DoubletFinder v0.2.0.4 package was used to remove doublets [88]. Gene expression count data for all samples was normalized with ‘NormalizedData’ function, followed by scaling to regress UMIs by ‘ScaleData’ function. Principal component analysis (PCA), UMAP, and tSNE implemented in the ‘RunPCA’, ‘RunUMAP’, and ‘RunTSNE’ functions were used to identify the deviations among cells. Differentially expressed genes (DEG) were identified by using the Wilcoxon rank-sum test implemented in the ‘FindMarkers’ function, and were considered significant with an average log_2_ fold change ≥ 0.25 and *P* adjusted < 0.05. Following DEG identification, “Prnp”, “APP695S”, and “Gh” were removed from plots as outliers. To generate heatmap visualization of microglial DAM and homeostatic DEGs identified from Keren-Shaul [48], the mean abundance for each transcript across all samples was set to 0 and the standard deviation was set to 1. This was accomplished in R for each gene of interest by subtracting the mean ‘RNA data’ of all samples from the mean ‘RNA data’ for each sample, and then dividing by the standard deviation across all samples. snRNA-seq data from this study has been deposited with Gene Expression Omnibus (GEO) (http://www.ncbi.nlm.nih.gov/geo/) under accession ID GSE292822.

### Behavioral analysis

Animals began behavioral testing between 9 and 12 mo of age for Vglut1-APP mice, or 23–24 mo for Gad2-APP. Animals were weighed and then handled for 5 min/day for 2 d prior to testing. Behavioral analysis began with open field (OF) on day 1, followed by straight swim (SS) on day 2, cued Morris water maze (MWM) on day 3, MWM training on day 4–8, and a single MWM probe test on day 11. Methods for OF, SS, and MWM were similar to [89]. SS and MWM testing was conducted in a circular tank measuring 58 cm high and 122 cm in diameter. The water level was approximately 20 cm from the top of the tank and made opaque using nontoxic white paint. Water temperature was maintained between 19 to 21 °C. Behavior studies were performed by a single experimenter blinded to genotype. Tracking of animal position was done with ANY-maze version 7.4 (Stoelting Co.). Between swim trials, animals were hand dried with a towel and returned to their home cage positioned half over a heating pad for warmth.

### Open field

OF testing was done in white acrylic open-top boxes (46 ×46 × 38 cm) under indirect lighting. Animals were allowed to explore for 30 min while movement was recorded from an overhead video camera.

### Straight swim

SS was used to acclimate animals to the water and assess swimming ability. A white, straight acrylic channel measuring 107 × 56 × 14 cm was placed in the center of the pool. Visible cues were removed from the room. Mice were allowed 60 seconds (sec) to reach a submerged platform on the opposite end of the channel. The platform (10 ×10 cm square) was covered in nylon mesh for traction and located 1–2 cm below the water surface. After 60 sec, mice that failed to reach the platform were guided to it by the experimenter. Mice stayed on the platform for 15 s before being removed from the tank, dried, and returned to their home cage. Mice were given 4 trials with 15–20 min intertrial intervals (ITI).

### Cued water maze (visible platform)

Following SS, mice were tested in a cued version of the water maze assess vision and check for stereotyped behaviors that would interfere with learning. Spatial cues were removed from the walls, and the platform was made visible by affixing a pole marked by black stripes. Mice were given 8 trials with a 15–20 min ITI. The platform was moved semi-randomly between trials to test each quadrant twice. Animals were placed into the pool from semi-random starting positions and allowed 60 sec to reach the platform where they were allowed to rest for 15 sec before being dried and placed back in their home cage.

### Morris water maze

Acquisition training in the MWM consisted of 4 trials per day with a 15–20 min ITI. Visible cues were placed on the walls to provide orientation. The hidden platform (10 ×10 cm square) was located in the northeast quadrant, half-way between the side and the center of the pool, 1–2 cm below the water surface. Mice were placed in the pool facing the wall at each of four randomized, cardinal start locations and allowed 60 sec to locate the platform. Animals that failed to locate the platform in the allotted time were guided there by the experimenter. Mice were allowed to stay on the platform for 15 sec before being returned to their home cage between trials. Mice were trained for 5 consecutive days. Long-term memory was tested three days after the final training trial by removing the platform and placing animals into the pool for a single probe test lasting 45 sec. Search error was used as a measure of performance and was automatically calculated in the AnyMaze software as cumulative distance from the zone. The measure is calculated from the animal’s distance from the platform multiplied by the time spent at that distance, summed across the duration of the trial.

### Statistical analyses

Statistical analyses were performed using GraphPad Prism 10.4.1. After testing normality by Shapiro-Wilk test, comparison of two groups was done using unpaired two-tailed Student’s t-test or Mann-Whitney for non-parametric distribution. Comparison of more than two groups was done using ANOVA followed by Tukey post-test. Comparison of two groups across days was done using repeated measures two-way ANOVA followed by Bonferroni post-hoc testing. Graphs were created using GraphPad and displayed as group means ± SEM.

### Figure preparation and writing

For all microscopy figures, images shown together were captured under the same exposure and illumination settings in Zeiss Zen Blue 2.6 and were adjusted identically in Photoshop 2022 for presentation, with the exception of Aβ (Ab9) staining in 24 mo Gad2-APP mice where the white point in Zen was lowered before export to match the signal intensity of 9 mo Vglut1-APP without altering the staining pattern (Figs. 2, 6, and 7). Text was written by the authors and edited with ChatGPT-4-omni (4o) or GPT-5.

## Electronic supplementary material

Below is the link to the electronic supplementary material.

Supplementary Material 1
