# Supplementary material for: Neuronal subtype governs amyloid structure, cellular response, and cognitive outcome in genetically targeted APP mouse models
Source: Mol Neurodegener. 2026 Jan 6;21:2. doi: 10.1186/s13024-025-00919-9 (PMC12790121; doi:10.1186/s13024-025-00919-9)
Supplement: Supplementary file 1 — Supplementary Material 1 [file 13024_2025_919_MOESM1_ESM.pdf]

Supplemental Data: Perez, Lai, et al.

Neuronal subtype governs amyloid structure, cellular response, and cognitive outcome in genetically targeted APP mouse models

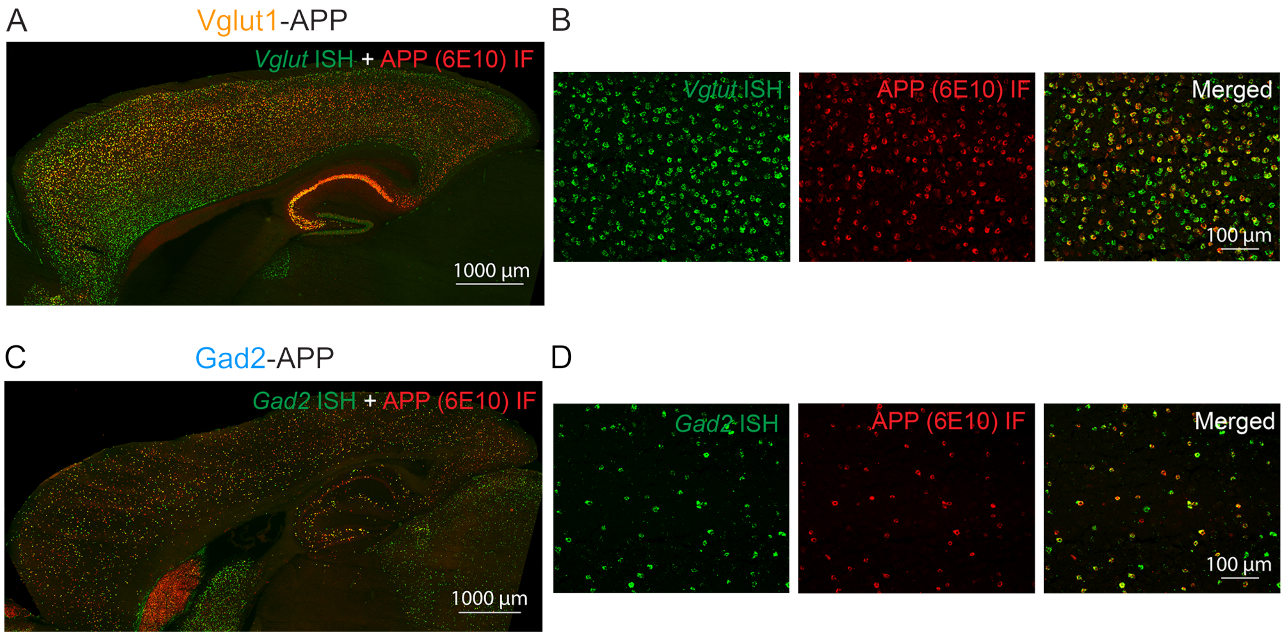

Supplemental Figure 1. Complementary patterns of transgenic APP expression in Vglut1-APP and Gad2-APP mice. (A) Representative brain section from a 1 mo Vglut1-APP mouse showing fluorescence in situ hybridization (ISH) to identify glutamatergic (Vglut1+2, green) neurons, followed by APP immunofluorescence (IF) (6E10, red) to detect transgenic APP+ cells. (B) High magnification view of Vglut1-APP cortex shows individual channels. (C) Representative brain section from a 1 mo Gad2-APP mouse showing ISH for Gad2 (green) followed by IF for transgenic APP (6E10, red). (D) High magnification view of Gad2-APP cortex shows individual channels. Both models were highly specific, with few APP+ cells that were not Vglut+ (A, B) or Gad2+ (C, D). However, neither model was completely efficient as occasional Vglut1+ or Gad2+ cells lacked APP expression. Images for each model were captured at different times and are not matched for exposure. Scale bar = 1 mm (A, C); 100 um (B, D)

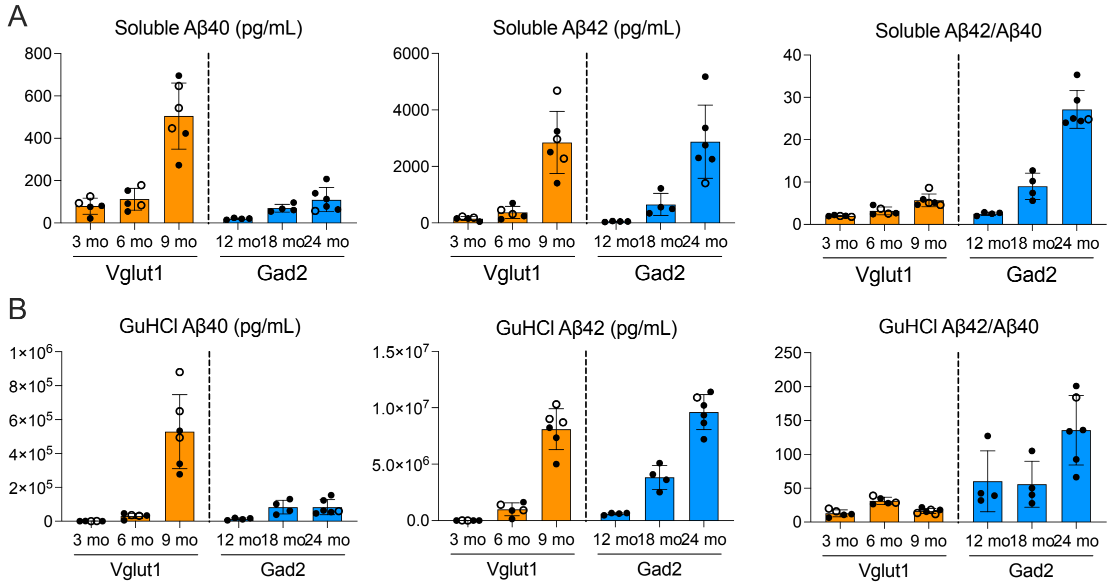

Supplemental Figure 2. Soluble and insoluble Aβ levels in cortical homogenates from aged Vglut1- and Gad2-APP mice. (A) TBS-EDTA soluble Aβ40 (left panel), Aβ42 (middle panel), and Aβ42/Aβ40 (right panel) at 3, 6, and 9 months for Vglut1-APP (orange bars) and 12, 18, and 24 months for Gad2-APP (blue bars). (B) Guanidine-soluble (i.e., TBS-insoluble) Aβ40 (left panel), Aβ42 (middle panel), and Aβ42/Aβ40 (right panel) at the same ages. n=4-6 mice per group (male – closed circles, female – open circles). Data presented as mean ± SEM.

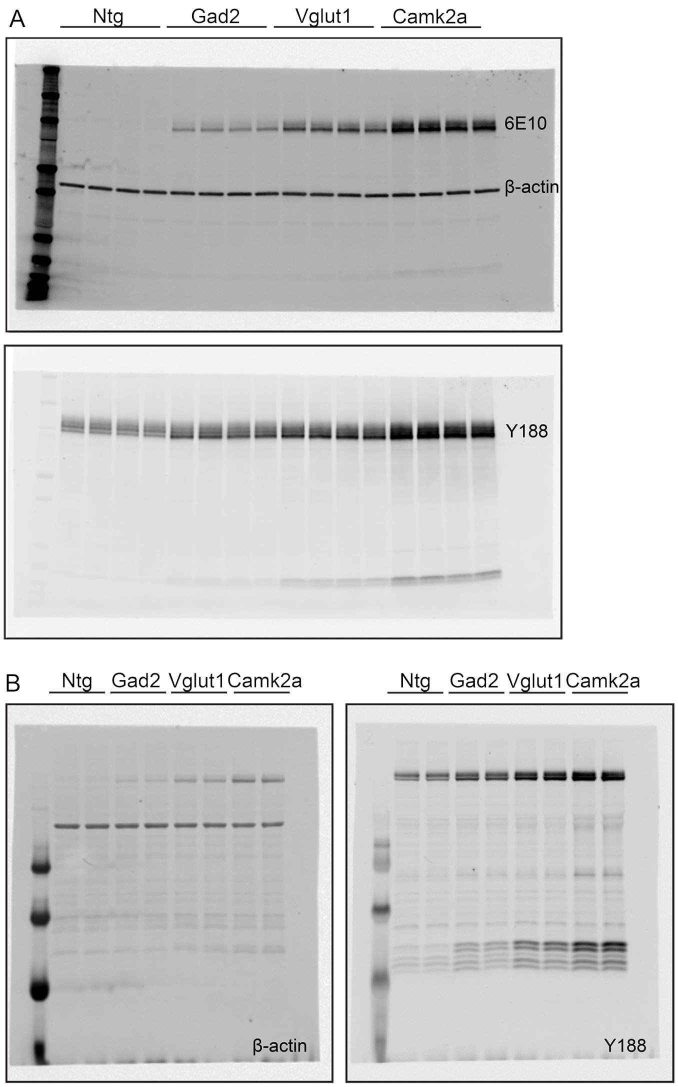

Supplemental Figure 3. Uncropped Western blots associated with Figure 5.

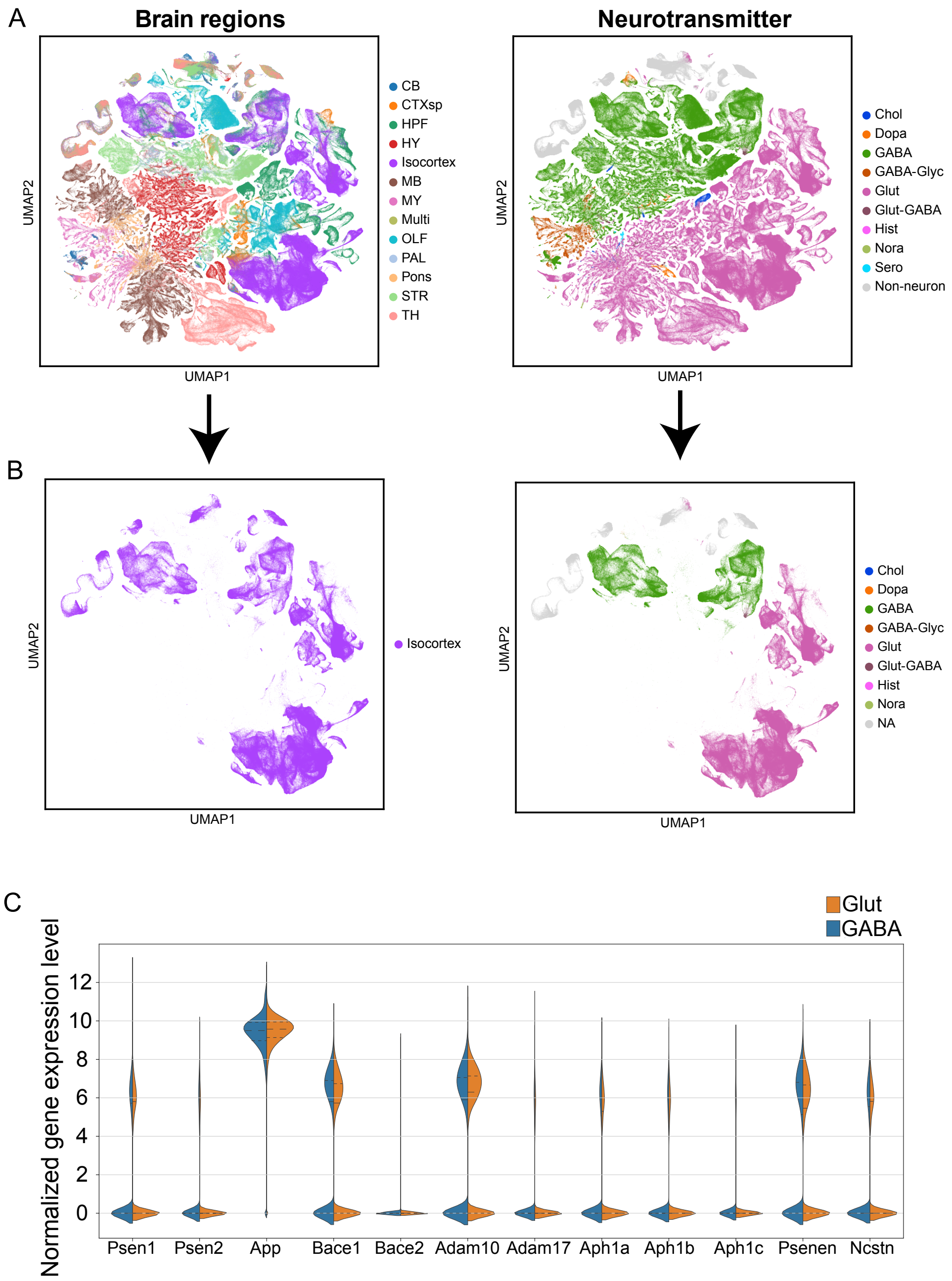

Supplemental Figure 4. No expression differences in key APP processing genes between excitatory and inhibitory neurons from wild-type mouse isocortex. (A) Uniform manifold approximation and projection (UMAP) representation of whole mouse brain single cell RNA sequencing (scRNA-seq) data showing 4,042,976 cells, annotated by brain region: CB, cerebellum; CTXsp, cortical subplate; HPF, hippocampal formation; HY, hypothalamus; Isocortex; MB, midbrain; MY, medulla; OLF, olfactory areas; P, pons; PAL, pallidum; STR, striatum; TH, thalamus (left panel) and by neurotransmitter type: Chol, cholinergic; Dopa, dopaminergic; GABA, GABAergic; Glut, glutamatergic; Glyc, glycinergic; Hist, histaminergic; Nora, noradrenergic; Sero, serotonergic; NA, not applicable (no neurotransmitter detected) (right panel). (B) UMAP of scRNA-seq data showing 1,451,483 cells identified as isocortex from whole mouse brain data, annotated by brain region (left panel) and neurotransmitter type (right panel). (C) Split violin plot showing expression level of APP processing genes in GABAergic (blue) and glutamatergic (orange) neurons from mouse isocortex. Dataset generated by Yao et al. (Yao et al., 2023) and obtained from the Allen Brain Cell Atlas. Data also available from Gene Expression Omnibus (GEO): GSE246717.

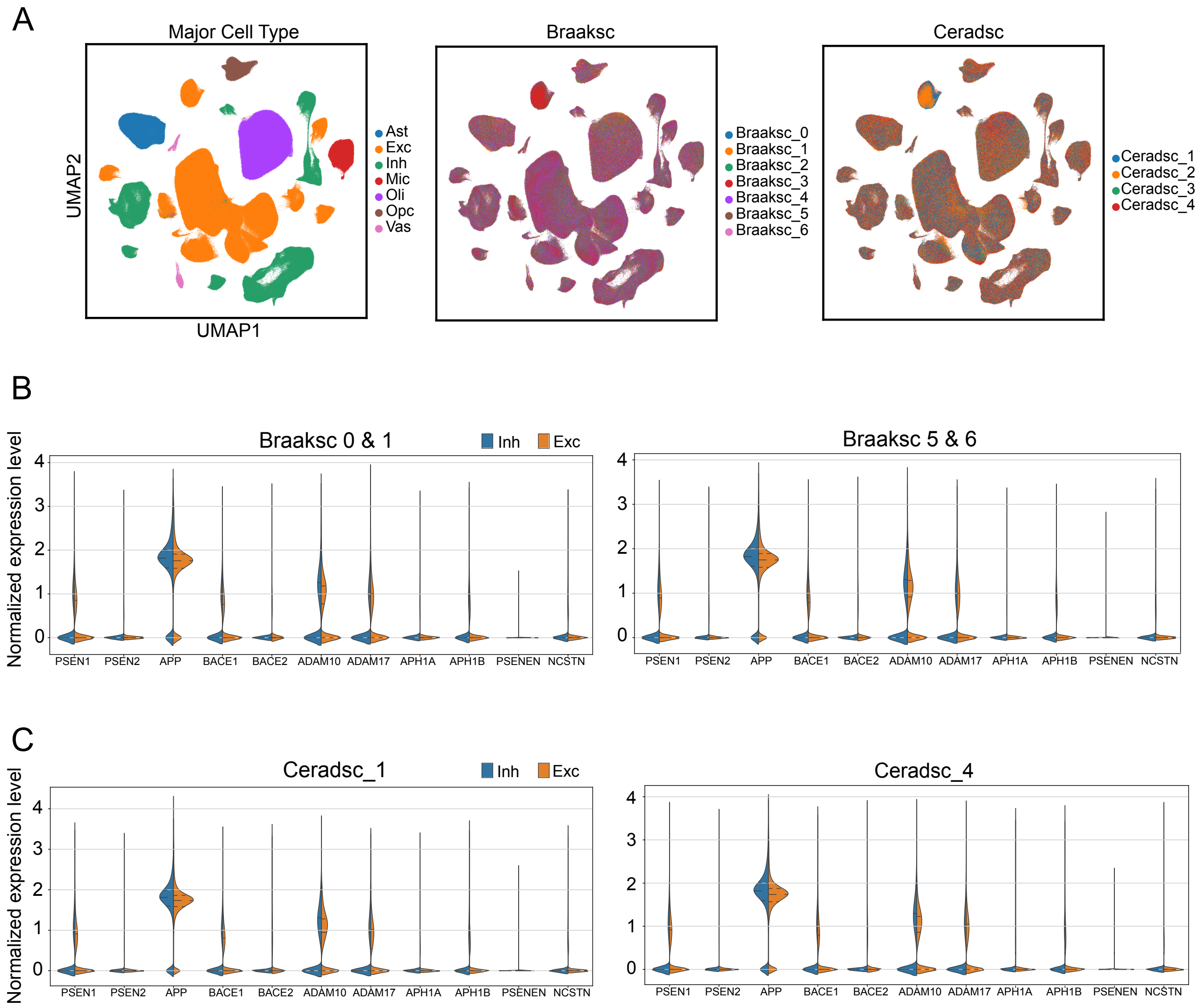

Supplemental Figure 5. No significant expression differences in APP processing genes between excitatory and inhibitory neurons from human prefrontal cortex. (A) UMAP of single nuclei RNA-seq (snRNA-seq) data showing 2,327,742 cells from 427 aged human prefrontal cortices annotated by major cell type (left panel), Braak stage (middle panel), and CERAD stage (right panel). (B) Split violin plots showing the expression level of APP processing genes in GABAergic (blue) and glutamatergic neurons (orange) for Braak 0 & 1 (left) and Braak 5 & 6 (right). (C) Split violin plots showing the expression level of APP processing genes in GABAergic (blue) and glutamatergic neurons (orange) for CERAD 1 (left) and CERAD 4 (right). Dataset generated by Mathys et al. (Mathys et al., 2023) and obtained from Synapse.org: syn52293417.

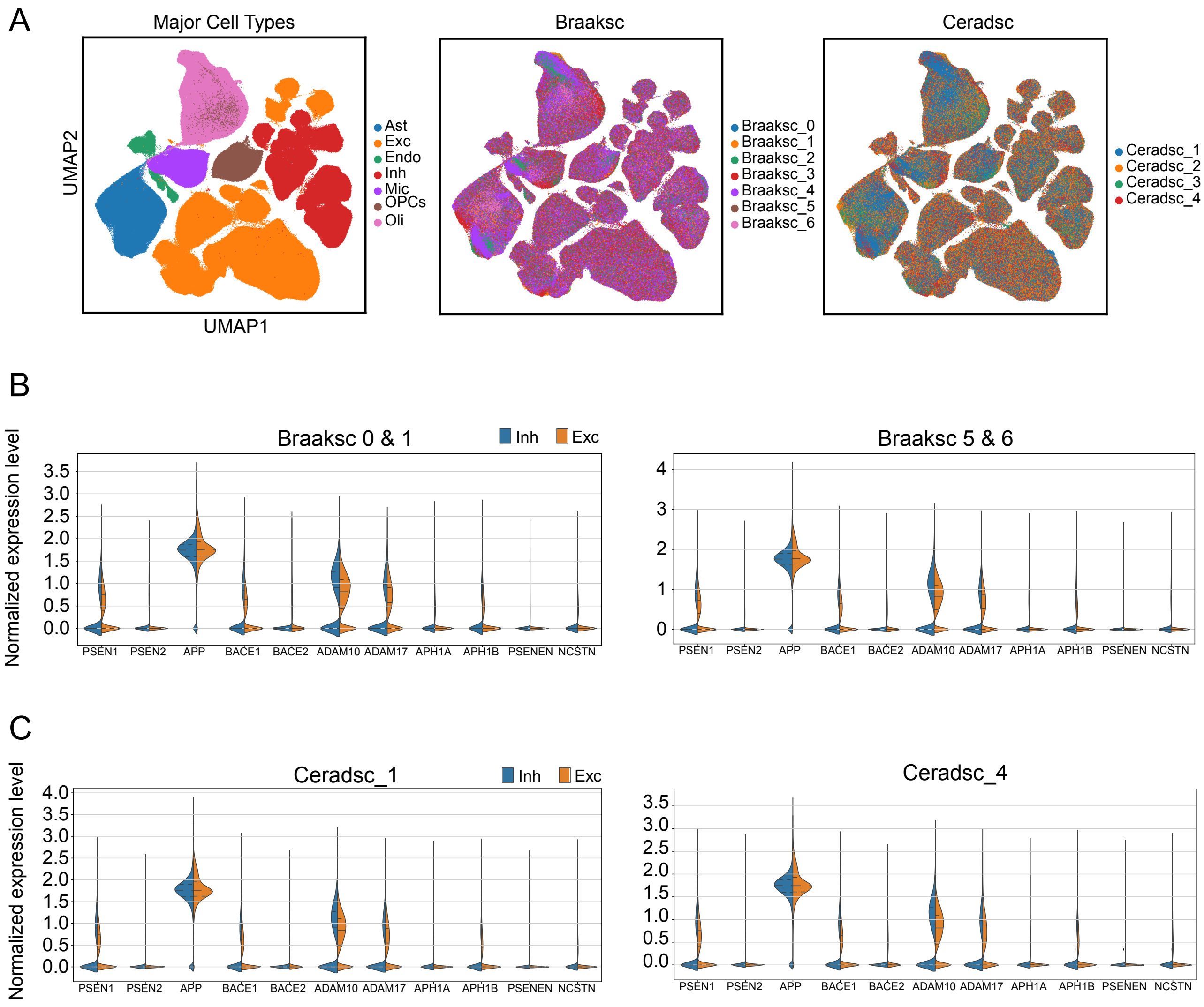

Supplemental Figure 6. No significant expression differences in APP processing genes between excitatory and inhibitory neurons from human dorsolateral prefrontal cortex. (A) UMAP representation of snRNA-seq data showing 1,649,672 cells from the neocortex of 424 individuals of advanced age annotated by major cell type (left panel), Braak stage (middle panel) and CERAD stage (right panel). (B) Split violin plots showing the expression level of APP processing genes in GABAergic (blue) and glutamatergic neurons (orange) for Braak 0 & 1 (left) and Braak 5 & 6 (right). (C) Split violin plots showing the expression level of APP processing genes in GABAergic (blue) and glutamatergic (orange) neurons for CERAD stage 1 (left) and CERAD stage 4 (right). Data generated by (Fujita et al., 2024) and obtained from Synapse.org: syn31512863.

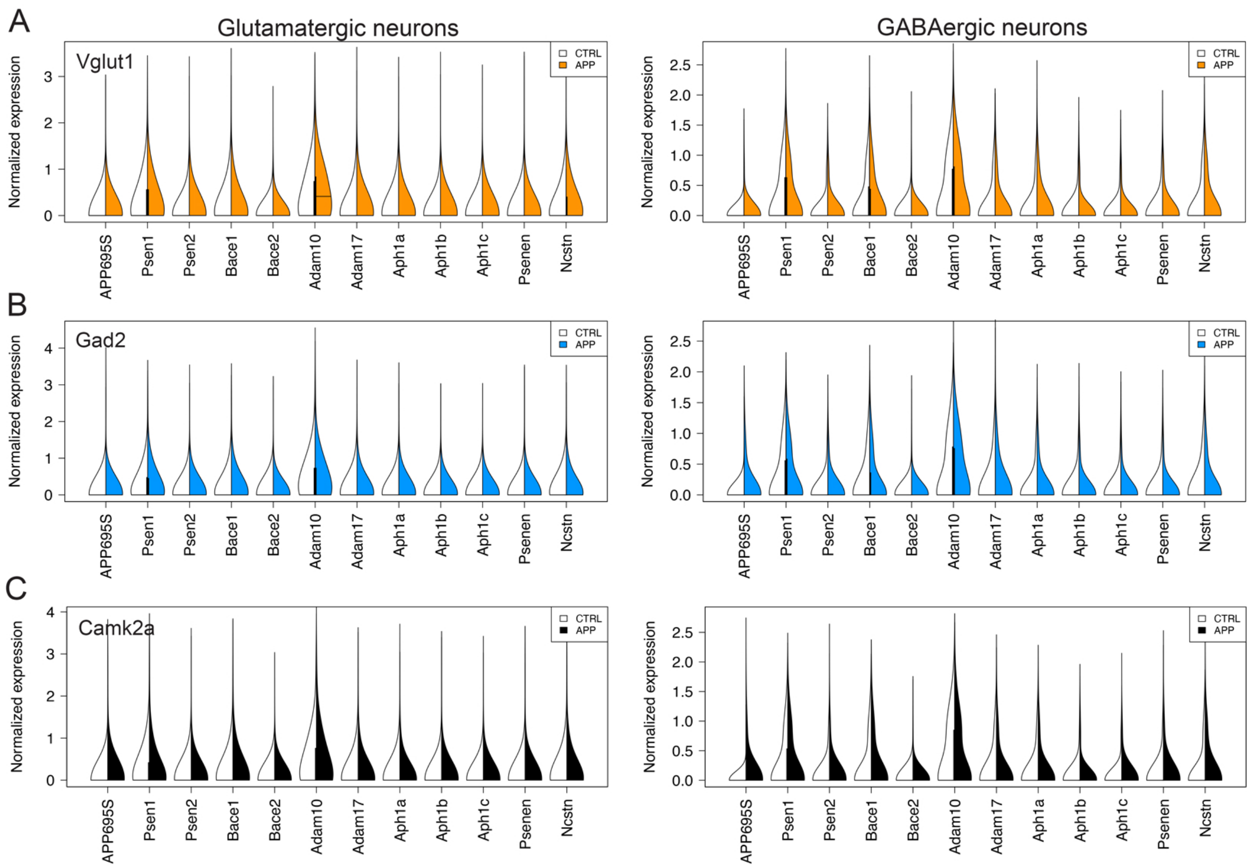

Supplemental Figure 7. No significant differences in mRNA expression of APP processing genes between excitatory and inhibitory neurons from Vglut1-, Gad2-, and Camk2a-APP studies. (A-C). Split violin plots for normalized expression of APP processing genes in rostral cortex snRNA-seq data from control (left, white outline) vs. APP transgenic mice (right, orange/blue/black) for excitatory (left panels) and inhibitory neurons (right panels). (A) 9 mo Vglut1-APP, (B) 24 mo Gad2-APP, and (C) 6 mo Camk2a-APP mice, each with age-matched control tissue.

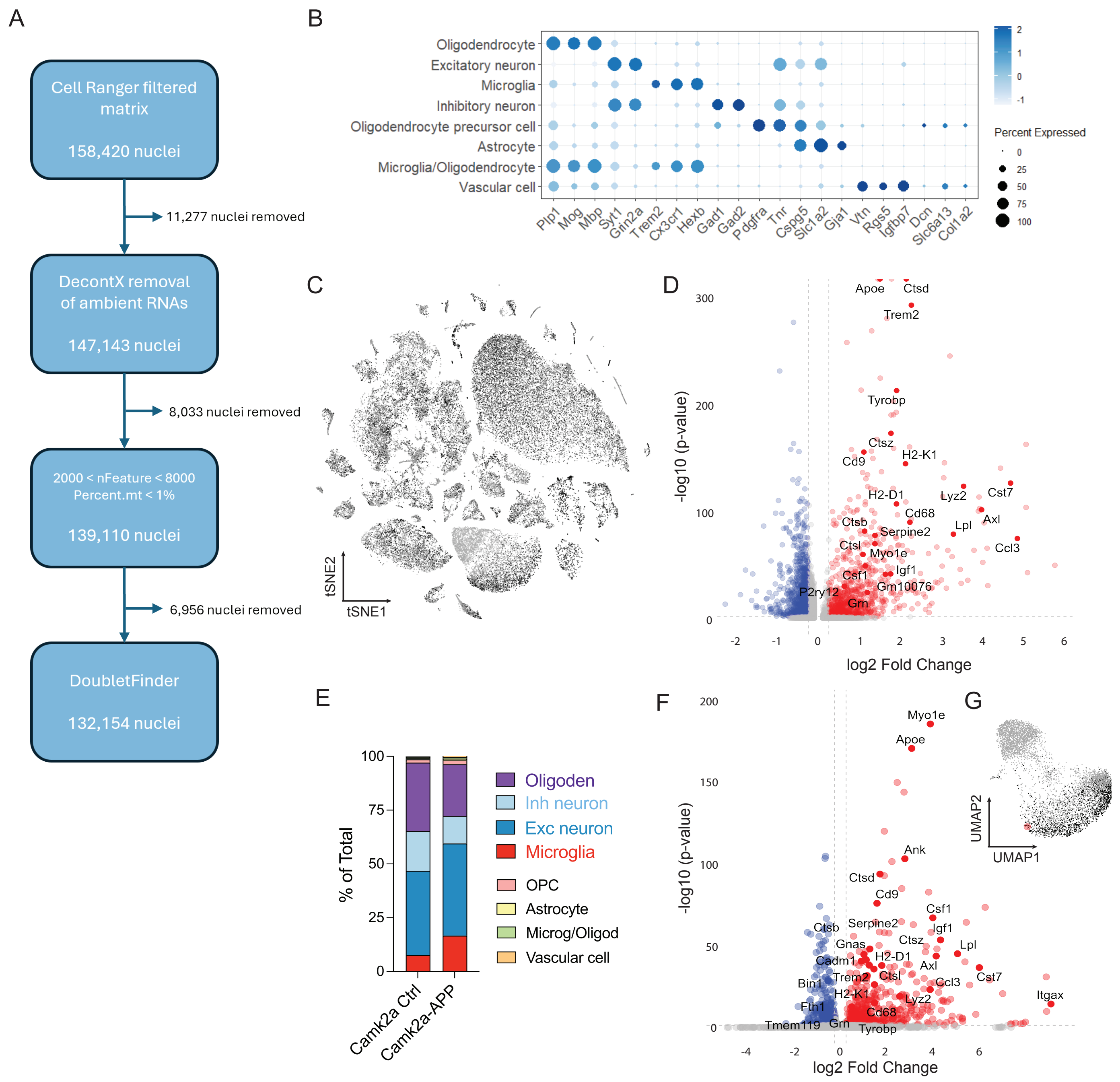

Supplemental Figure 8. snRNA-seq analysis of rostral cortex from Vglut1-APP (9 mo), Gad2-APP (24 mo), and Camk2a-APP (6 mo) mice. (A) Total number of nuclei removed and remaining after each step of quality control processing. (B) Dot plot showing expression of mRNAs used to classify each cell type. Intensity reflects mean expression level of the gene of interest, and size reflects percentage that cluster expressing the gene. (C) t-distributed stochastic neighbor embedding (tSNE) plot of 45,743 nuclei colored by genotype for 6 mo Camk2a-APP (grey) and control samples (black). (D) Volcano plot showing significantly up- and down-regulated genes for all cell types between Camk2a-APP and controls. Labeled points on this and subsequent volcano plots identify significantly altered markers of disease-associated microglia. Wilcoxon rank-sum test, adjusted p-value ≤ 0.05, $\log_2$ FC > 0.25 (E) Stacked bar graph showing proportion of each cell type identified by genotype. (F) Volcano plot showing significantly up- and down-regulated microglial genes between Camk2a-APP and controls. (G) UMAP plot of 5,553 microglial nuclei colored by genotype for Camk2a-APP (grey) and controls samples (black).

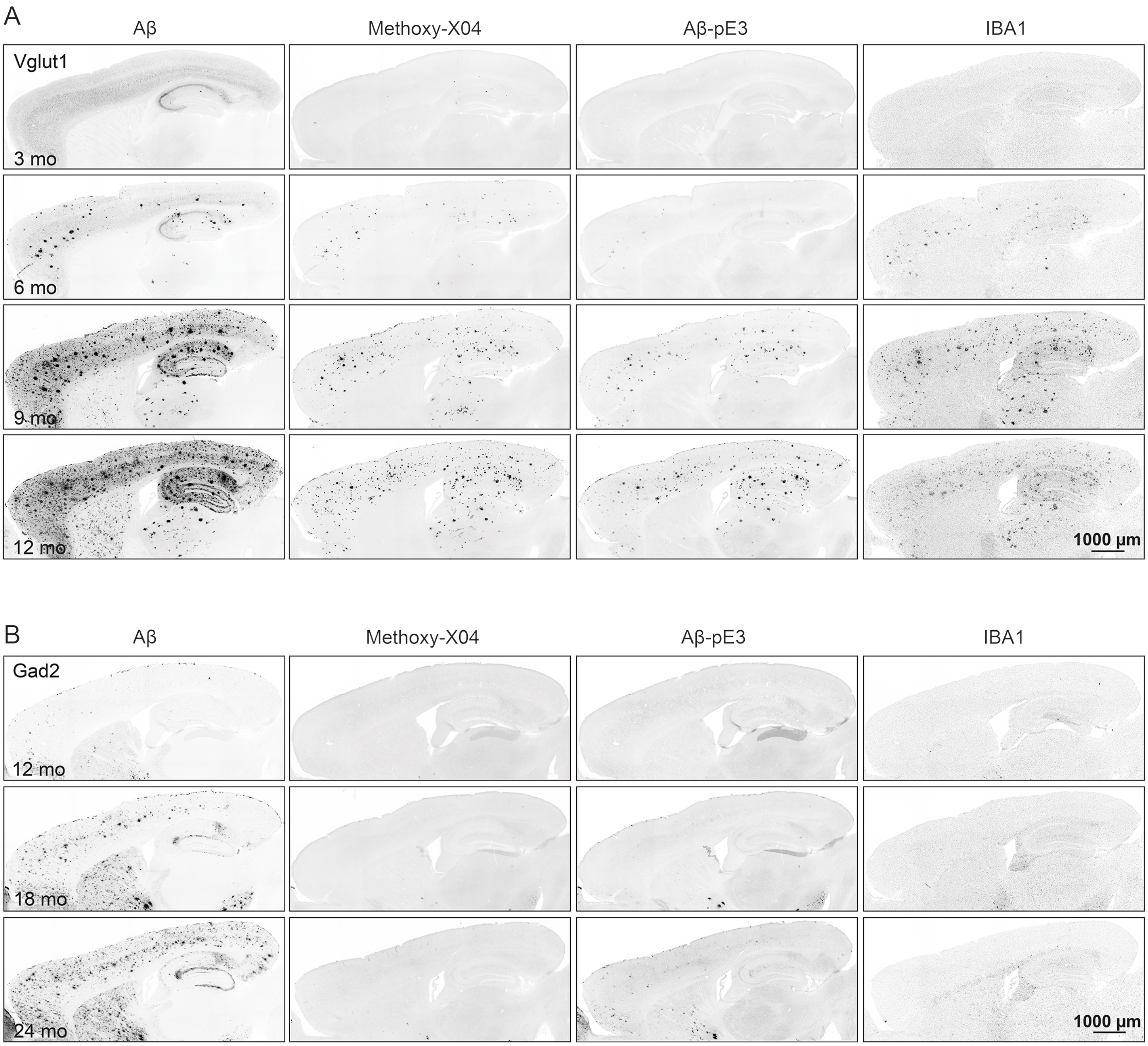

Supplemental Figure 9. Pyroglutamate-3 Aβ is restricted to fibrillar deposits made by Vglut1-APP mice, but appears after plaques are detected with methoxy-X04 and are surrounded by microgliosis. Representative brain sections from Vglut1-APP mice at 3, 6, 9, and 12 months (A) and Gad2-APP mice at 12, 18, and 24 months (B), immunostained for Aβ (human-specific clone Ab9), methoxy-X04, pyroglutamate-3 Aβ (Aβ-pE3), and IBA1. Aβ, methoxy-X04, and Aβ-pE3 were co-stained on the same section; IBA1 was done separately using an adjacent section. Scale bar = 1 mm

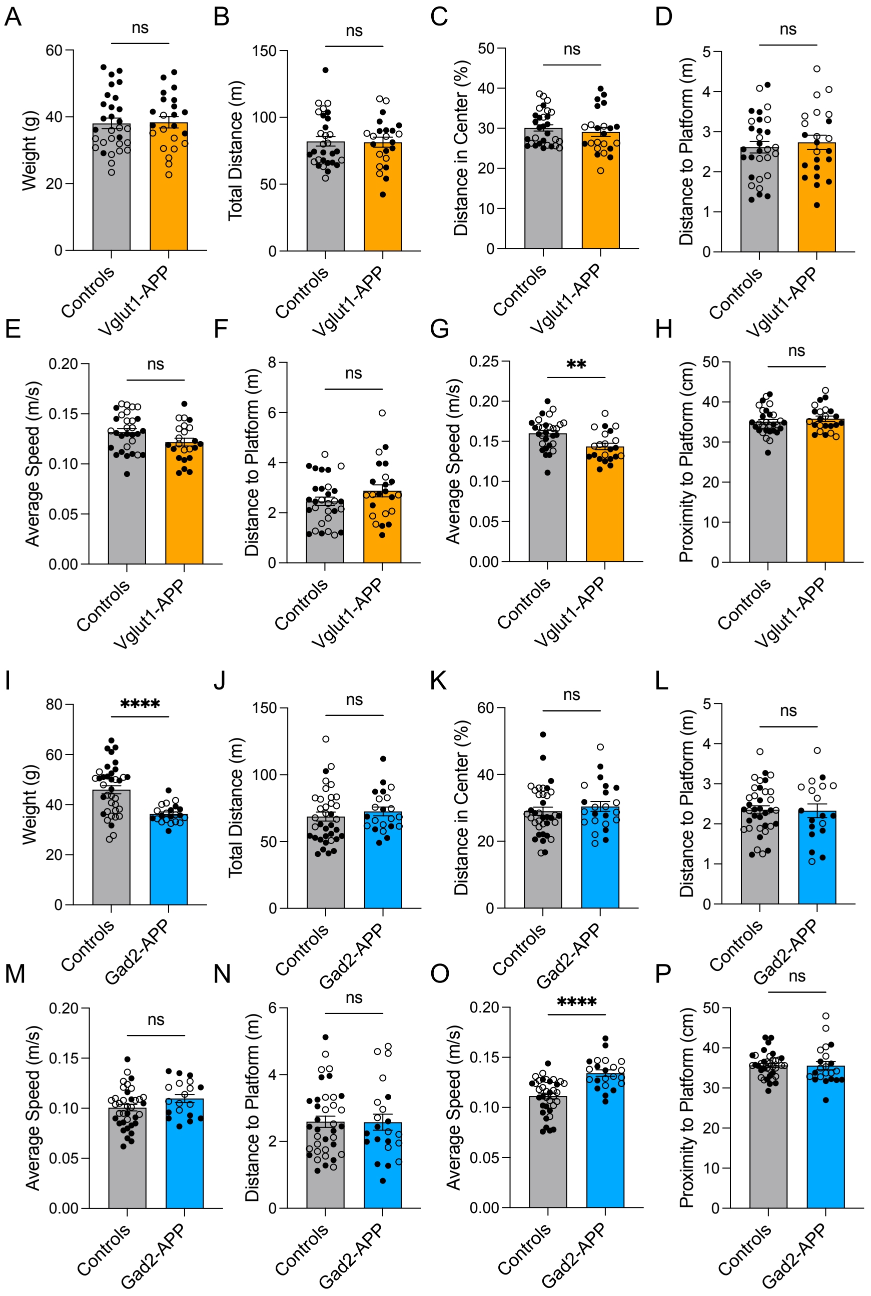

Supplemental Figure 10. APP and control mice performed similarly in most measures of locomotor ability, visual acuity, and anxiety. (A-H) Behavior measurements for 9-12 mo Vglut1-APP and control mice. (A) Body weight at the start of testing. (B) Open field total distance traveled over the single 30 min trial. (C) Open field percent of total distance traveled in the center portion of the arena. (D) Straight swim average total distance traveled to the escape platform across four 60 sec trials. (E) Straight swim average swim speed. (F) Cued MWM average distance to escape platform over eight 60 sec trials. (G) Cued MWM average swim speed. (H) Cued MWM average distance to the escape platform. (I-P) Behavior measurements for 23-24 mo Gad2-APP and control mice. (I) Body weight at the start of testing. (J) Open field total distance traveled over the single 30 min trial. (K) Open field percent of total distance traveled in the center portion of the arena. (L) Straight swim average total distance traveled to the escape platform across four 60 sec trials. (M) Straight swim average swim speed. (N) Cued MWM average distance to escape platform over eight 60 sec trials. (O) Cued MWM trials average swim speed. (P) Cued MWM average distance to the escape platform. n=30 Vglut1 controls (wild-type or Cre+/tTA+/APP-), 23 Vglut1-APP, 37 Gad2 controls (wild-type or Cre+/tTA+/APP-), and 22 Gad2-APP. (male – closed circles; female – open circles).Data shown as mean ± SEM. Unpaired two-tailed t-test (A, D-O), Mann-Whitney two-tailed test (B, C, P)

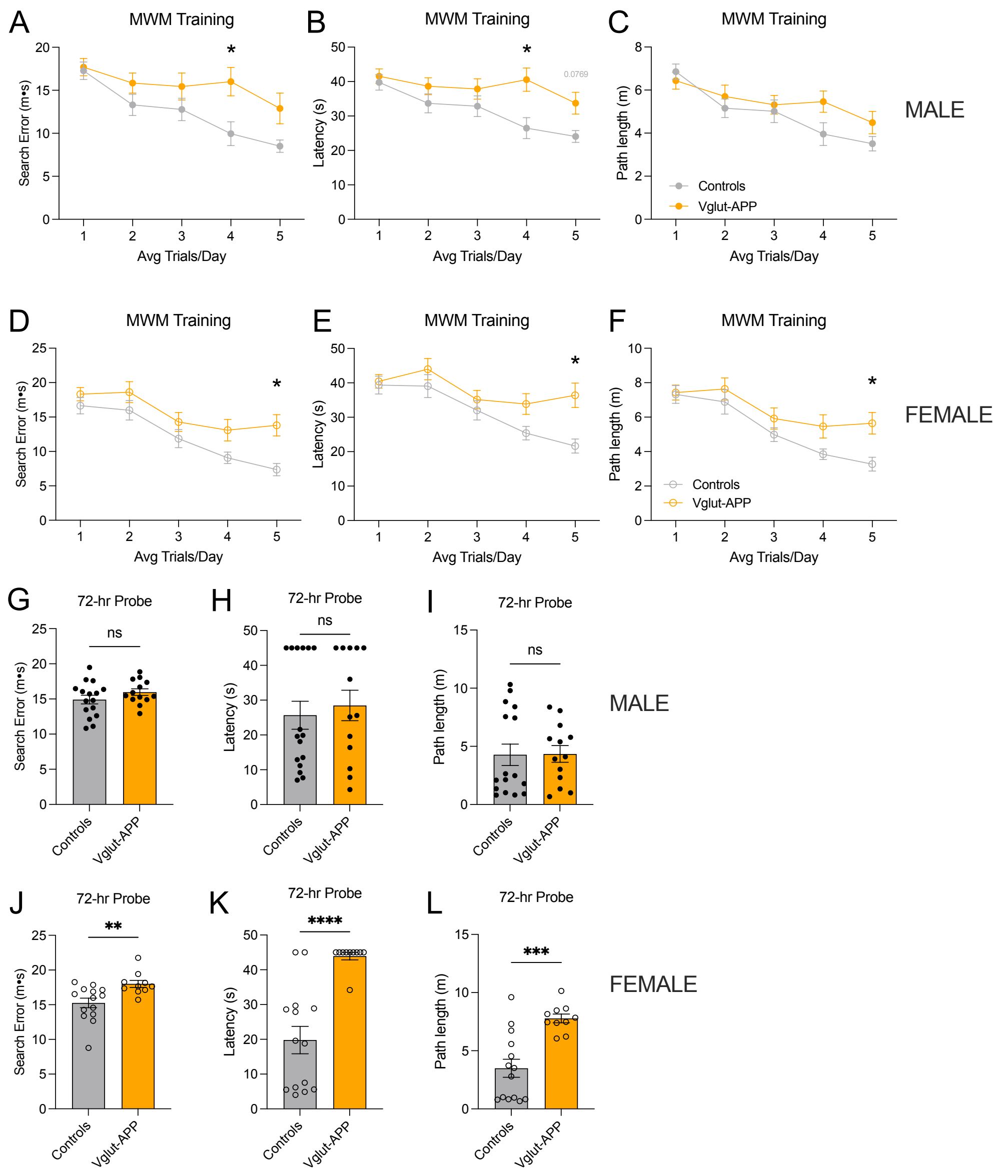

Supplemental Figure 11. Morris water maze performance for Vglut-APP mice, separated by sex. (A-F) Performance was assessed as proximity to the platform (search error, A, D), escape latency (B, E), and path length (C, F). Data for males is shown in A-C, females in D-F. (G-L) Performance during probe trials was assessed as proximity (G, J), latency (H, K), and path length to the trained location (I, L). Data for males is shown in G-I, females in J-L. Males: n = 16 Vglut1 controls (wild-type or Cre+/tTA+/APP-), and 13 Vglut1-APP. Females: n = 14 Vglut1 controls (wild-type or Cre+/tTA+/APP-), and 10 Vglut1-APP. Data are shown as mean ± S.E.M. Two-way repeated measure ANOVA with Bonferroni posttest (A-F), unpaired two-tailed t-test (G, J), Mann-Whitney two-tailed test (H, I, K, L); ns = non-significant, *$p<0.05$, **$p<0.01$, ***$p<0.001$, ****$p<0.0001$

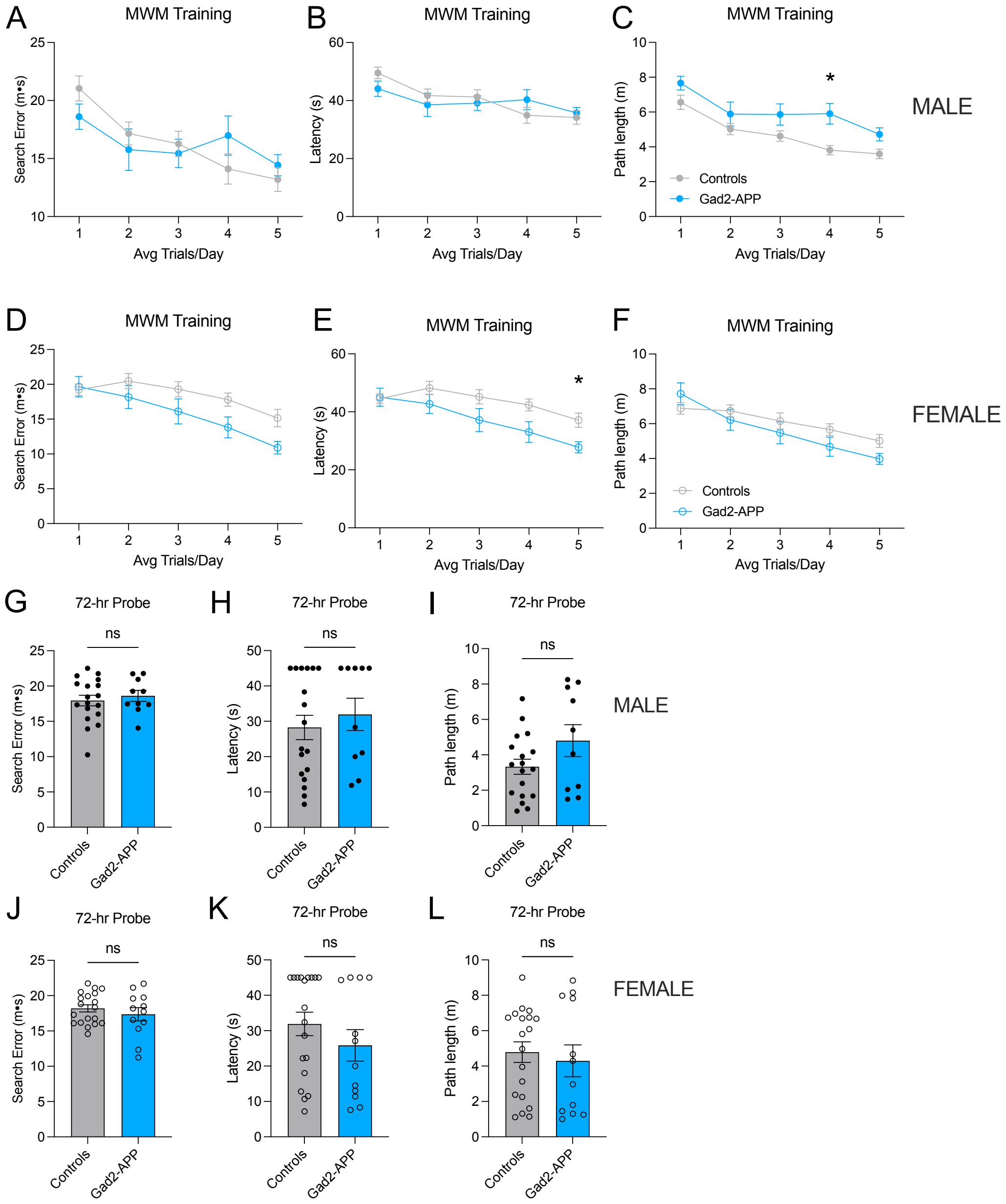

Supplemental Figure 12. Morris water maze performance for Gad2-APP mice, separated by sex. (A-F) Performance was assessed as proximity to the platform (search error, A, D), escape latency (B, E), and path length (C, F). Data for males is shown in A-C, females in D-F. (G-L) Performance during probe trials was assessed as proximity (G, J), latency (H, K), and path length to the trained location (I, L). Data for males is shown in G-I, females in J-L. Males: n = 18 Gad2 controls (wild-type or Cre+/tTA+/APP-), and 10 Gad2-APP. Females: n = 19 Gad2 controls (wild-type or Cre+/tTA+/APP-), and 12 Gad2-APP. Data are shown as mean ± S.E.M. Two-way repeated measure ANOVA with Bonferroni posttest (A-F), unpaired two-tailed t-test (G, I, J), Mann-Whitney two-tailed test (H, K, L); ns = non-significant, *$p<0.05$.

References

Fujita, M., Gao, Z., Zeng, L., McCabe, C., White, C.C., Ng, B., Green, G.S., Rozenblatt-Rosen, O., Phillips, D., Amir-Zilberstein, L., *et al.* (2024). Cell subtype-specific effects of genetic variation in the Alzheimer's disease brain. Nat Genet 56, 605-614.

Mathys, H., Peng, Z., Boix, C.A., Victor, M.B., Leary, N., Babu, S., Abdelhady, G., Jiang, X., Ng, A.P., Ghafari, K., *et al.* (2023). Single-cell atlas reveals correlates of high cognitive function, dementia, and resilience to Alzheimer's disease pathology. Cell 186, 4365-4385 e4327.

Yao, Z., van Velthoven, C.T.J., Kunst, M., Zhang, M., McMillen, D., Lee, C., Jung, W., Goldy, J., Abdelhak, A., Aitken, M., *et al.* (2023). A high-resolution transcriptomic and spatial atlas of cell types in the whole mouse brain. Nature 624, 317-332.
